# Chromosome 8p engineering reveals increased metastatic potential targetable by patient-specific synthetic lethality in liver cancer

**DOI:** 10.1126/sciadv.adh1442

**Published:** 2023-12-22

**Authors:** Thorben Huth, Emely C. Dreher, Steffen Lemke, Sarah Fritzsche, Raisatun N. Sugiyanto, Darko Castven, David Ibberson, Carsten Sticht, Eva Eiteneuer, Anna Jauch, Stefan Pusch, Thomas Albrecht, Benjamin Goeppert, Julián Candia, Xin Wei Wang, Junfang Ji, Jens U. Marquardt, Sven Nahnsen, Peter Schirmacher, Stephanie Roessler

**Affiliations:** ^1^ Heidelberg University, Medical Faculty, Institute of Pathology, University Hospital Heidelberg, 69120 Heidelberg, Germany.; ^2^ Quantitative Biology Center (QBiC), University of Tübingen, 72076 Tübingen, Germany.; ^3^ Department of Peptide-based Immunotherapy, University and University Hospital Tübingen, 72076 Tübingen, Germany.; ^4^ Institute for Cell Biology, Department of Immunology, University of Tübingen, 72076 Tübingen, Germany.; ^5^ Cluster of Excellence iFIT (EXC2180) “Image-Guided and Functionally Instructed Tumor Therapies”, University of Tübingen, 72076 Tübingen, Germany.; ^6^ Department of Medicine I, University Medical Center Schleswig Holstein, 23538 Lübeck, Germany.; ^7^ Deep Sequencing Core Facility, CellNetworks Excellence Cluster, Heidelberg University, 69120 Heidelberg, Germany.; ^8^ NGS Core Facility, Medical Faculty Mannheim, Heidelberg University, 68167 Mannheim, Germany.; ^9^ Institute of Human Genetics, Heidelberg University, 69120 Heidelberg, Germany.; ^10^ Department of Neuropathology, Institute of Pathology, University Hospital Heidelberg, 69120 Heidelberg, Germany.; ^11^ Clinical Cooperation Unit Neuropathology, German Cancer Research Center (DKFZ), 69120 Heidelberg, Germany.; ^12^ Institute of Tissue Medicine and Pathology, University of Bern, 3008 Bern, Switzerland.; ^13^ Institute of Pathology and Neuropathology, RKH Klinikum Ludwigsburg, 71640 Ludwigsburg, Germany.; ^14^ Longitudinal Studies Section, Translational Gerontology Branch, National Institute on Aging, National Institutes of Health, Baltimore, MD 21224, USA.; ^15^ Laboratory of Human Carcinogenesis and Liver Cancer Program, Center for Cancer Research, National Cancer Institute, National Institutes of Health, Bethesda, MD 20892, USA.; ^16^ The MOE Key Laboratory of Biosystems Homeostasis & Protection, Zhejiang Provincial Key Laboratory for Cancer Molecular Cell Biology, and Innovation Center for Cell Signaling Network, Life Sciences Institute, Zhejiang University, Hangzhou 310058, China.; ^17^ Biomedical Data Science, Department of Computer Science, University of Tübingen, 72076 Tübingen, Germany.; ^18^ The M3 Research Center, University of Tübingen, 72076 Tübingen, Germany.

## Abstract

Large-scale chromosomal aberrations are prevalent in human cancer, but their function remains poorly understood. We established chromosome-engineered hepatocellular carcinoma cell lines using CRISPR-Cas9 genome editing. A 33–mega–base pair region on chromosome 8p (chr8p) was heterozygously deleted, mimicking a frequently observed chromosomal deletion. Using this isogenic model system, we delineated the functional consequences of chr8p loss and its impact on metastatic behavior and patient survival. We found that metastasis-associated genes on chr8p act in concert to induce an aggressive and invasive phenotype characteristic for chr8p-deleted tumors. Genome-wide CRISPR-Cas9 viability screening in isogenic chr8p-deleted cells served as a powerful tool to find previously unidentified synthetic lethal targets and vulnerabilities accompanying patient-specific chromosomal alterations. Using this target identification strategy, we showed that chr8p deletion sensitizes tumor cells to targeting of the reactive oxygen sanitizing enzyme Nudix hydrolase 17. Thus, chromosomal engineering allowed for the identification of novel synthetic lethalities specific to chr8p loss of heterozygosity.

## Teaser

Chromosomes were engineered to mimic alterations observed in cancer patients to better understand tumor development and treatment.

## INTRODUCTION

Chromosomal alterations, genomic instability, and aneuploidy are a hallmark of cancer (*1*). Up to 88% of human cancers harbor arm-level chromosomal alterations that, on average, affect one-fourth of their whole genome (*2*, *3*). Despite this high frequency, only a minority of deletions can be explained by loss of one single tumor suppressor gene fulfilling the two-hit hypothesis criteria (*4*, *5*). Cumulative haploinsufficiency has been described as a potential hypothesis for enrichment of some chromosomal deletions in cancer. Hereby, the loss of cooperating tumor suppressor genes provides a proliferative advantage. Still, cumulative haploinsufficiency may not only affect proliferation and survival genes but also affect other aspects of tumorigenesis such as drug resistance or metastasis (*6*). Consistently, deletion of single-chromosome arms and increased aneuploidy are associated with poor patient prognosis in solid cancers (*7*–*9*).

In hepatocellular carcinoma (HCC), a median of eight chromosome arm-level aneuploidies is detected per tumor (*7*). The most frequent aberrations encompass gain of 1q, 8q, 17q, or 20q and loss of 4q, 8p, 13q, 16q, or 17p (*10*, *11*). These alterations have been shown to be caused by chromosomal instability which is thought to affect HCC development and aggressiveness (*12*, *13*). Among those arm-level alterations, chromosome 8p (chr8p) loss of heterozygosity (LOH) is one of the most frequent deletions in solid tumors and has been shown to decrease overall patient survival in HCC (*14*). LOH refers here to the heterozygous deletion of chr8p and does not include copy number neutral LOH. To date, several tumor suppressor genes on chr8p have been described, but analysis of individual genes failed to account for the high prevalence of chr8pLOH. In addition, cosuppression of multiple chr8p genes has been described to show antiproliferative effects and affect cell metabolism (*15*–*17*). On the other hand, clinical studies have identified increased occurrence of metastasis in patients with chr8pLOH (*18*, *19*). However, the underlying mechanisms remain poorly understood and cannot be assigned to single gene losses.

Aside from the competitive advantages, large-scale aberrations can lead to acquired vulnerabilities through the concomitant loss of passenger genes enabling identification of potent treatment strategies (*20*). The investigation of these accessory effects of large-scale chromosomal deletions has been mostly limited to studies of a small number of candidate genes (*16*). In addition, not all murine orthologs of the human chr8p genes are located on chr8p of mice making it unfeasible to study chr8p deletion in mouse models (*21*). CRISPR-Cas9 accelerated the development of a plethora of different gene-editing technologies, which opens the possibility to genetically engineer arm-level deletions of chromosomes and dissect their acquired vulnerabilities (*22*). Exploitation of chromosome-engineered cell lines allows reflecting the complexity of large-scale copy number aberrations in tumors granting a more holistic view on the effects of arm-level deletions and is a particularly suited tool for the identification of previously unknown vulnerabilities.

Here, we describe the engineering of three different HCC cell lines with heterozygous loss of chr8p and its further use as a platform to identify functionally relevant genes and previously unidentified vulnerabilities acquired by the arm-level deletion of chr8p. These isogenic cell lines served as powerful tools, and we demonstrated that chr8pLOH led to enhanced metastatic behavior. Furthermore, using a whole-genome CRISPR-Cas9 screening approach, we identified the Nudix (nucleoside diphosphate–linked moiety X) hydrolases NUDT17 and NUDT18 as synthetic lethal paralogs, suggesting that NUDT17 may be targetable in liver cancer cells with chr8pLOH.

## RESULTS

### chr8p loss is associated with down-regulation of multiple tumor suppressor gene candidates

**Fig. 1.**
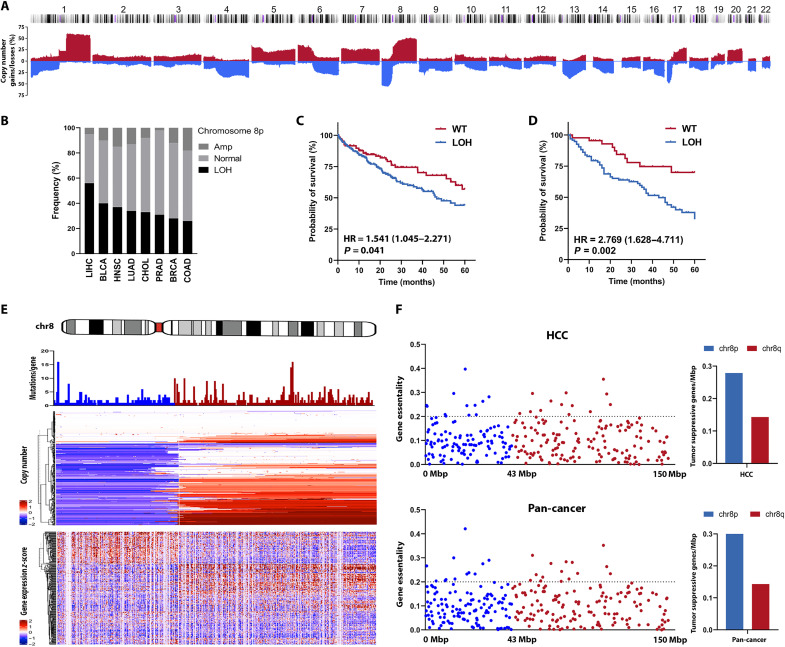
chr8pLOH is a frequent event observed in a plethora of cancer entities and associated with poor outcome. (**A**) Copy number variation profile of patients with HCC from the TCGA-LIHC cohort visualized by progenetix.org. (**B**) Frequency of chr8p copy number alterations in different cancer entities. chr8pLOH groups were defined by a mean copy number of <−0.33 in LIHC (HCC), BLCA (bladder urothelial carcinoma), HNSC (head and neck squamous cell carcinoma), LUAD (lung adenocarcinoma), CHOL (cholangiocarcinoma), PRAD (prostate adenocarcinoma), BRCA (breast invasive carcinoma), and COAD (colon adenocarcinoma). (**C**) Kaplan-Meier survival curves of patients with TCGA-LIHC and (**D**) LICA-FR clustered into chr8p wild-type (WT) [red; *N* = 117 (C) and *N* = 52 (D)] or chr8pLOH [blue; *N* = 227 (C) and *N* = 100 (D)] according to mean copy number. Hazard ratio (HR) with 95% confidence interval and *P* values were calculated by log-rank test. (**E**) Mutation, copy number, and gene expression data of chr8 in the TCGA-LIHC cohort. Numbers of missense and nonsense mutations per gene are shown. Genes on chr8p are colored in blue, and those on chr8q are colored in red (top). Clustered heatmap visualization of copy number variation on chr8. Deletions are colored in blue, and amplifications are colored in red (center). Visualization of *z*-scores of chr8 gene expression (bottom). (**F**) Gene essentiality scores for chr8p (blue) and chr8q genes (red) in liver cancer cell lines (top) or in solid tumor cell lines (pan-cancer; bottom) obtained from Broad Institute’s DepMap database. Only genes with positive gene essentiality scores are depicted. Quantification of genes with essentiality scores of >0.2 per Mbp.

Examination of The Cancer Genome Atlas (TCGA) datasets revealed high copy number losses affecting large chromosome stretches in many solid tumor entities (Fig. 1, A and B, and fig. S1, A to F). Among those, chr8pLOH is one of the most predominant arm-level losses in tumor entities including liver, lung, pancreas, breast, and colon cancer (Fig. 1B). Analysis of the overall survival time of patients harboring chr8p deletions underlined the clinical relevance of this chromosome arm deletion. Patients harboring a chr8p loss of at least 33% of the chromosome arm according to copy number data were compared to patients with only minor copy number alterations affecting less than 10% of the chromosome arm. The resulting Kaplan-Meier curves indicated that chr8pLOH significantly decreased the overall patient survival especially for liver, breast, and head and neck cancer (Fig. 1C and fig. S1, G to L). Furthermore, we validated the association of chr8pLOH with worse overall survival in an independent publicly available cohort of patients with HCC (LICA-FR; Fig. 1D) (*23*). This emphasized the need for a more thorough understanding of the molecular mechanisms underlying chr8pLOH. As heterozygous chr8p loss is most prevalent in liver cancer, affecting more than half of all patients with HCC (Fig. 1, A and B), we further investigated mutation status and gene expression in the TCGA-LIHC dataset (Fig. 1E). Although many patients harbor deletions, often spanning the whole chr8p arm, no major mutation hotspot indicating a tumor suppressor according to Knudson’s two-hit hypothesis was identified (Fig. 1E). Similarly, gene expression analysis revealed uniform down-regulation of chr8p genes in patients with chr8pLOH (Fig. 1E, bottom). As determined in CRISPR-Cas9 viability screens of the Cancer Dependency Map Portal (DepMap; https://depmap.org/portal/) (*24*), loss of tumor suppressor genes increased cell viability indicated by positive gene essentiality scores. We found that the number of genes with a gene essentiality score of >0.2 per mega–base pair (Mbp) is twice as high on chr8p compared to the frequently amplified chr8q in liver cancer and pan-cancer (Fig. 1F). This suggested that chr8pLOH may be selected by cumulative haploinsufficiency of multiple tumor suppressive genes accounting for poor overall patient survival.

### Generation of chromosome-engineered chr8pLOH cell lines

**Fig. 2.**
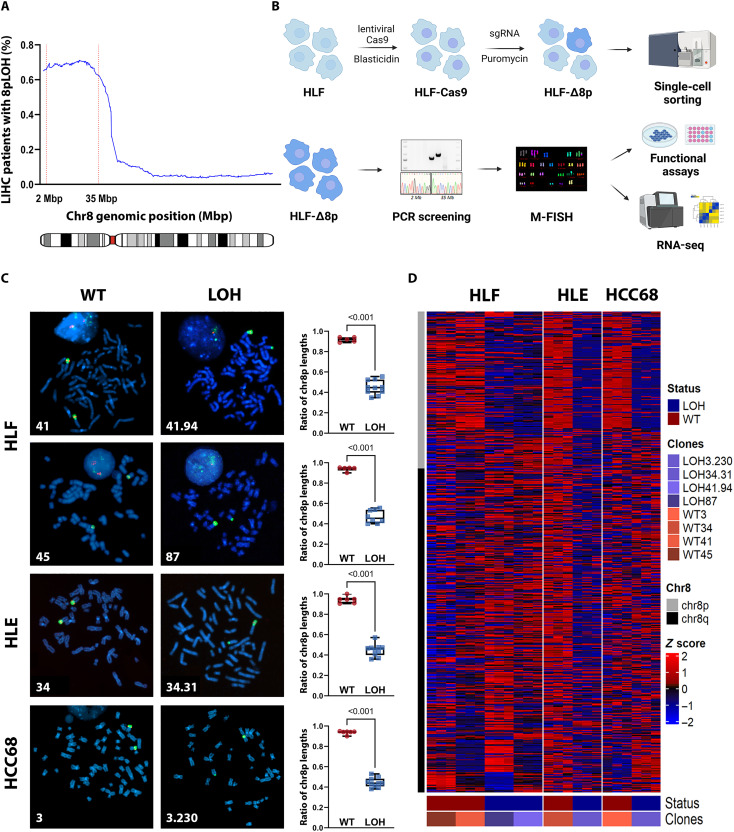
Engineering of chr8p loss in HCC cells by CRISPR-Cas9 technology and validation of chr8pLOH-harboring clones. (**A**) Shifting window plot for percentage of LOH at different genomic locations on chr8. The 2- and 35-Mbp cut sites are indicated by dotted vertical lines with chr8pLOH of >60%. (**B**) Workflow for engineering and validation of chr8pLOH cell lines. Images were created with biorender.com. (**C**) FISH of metaphase chr8pWT and chr8pLOH cell clones staining the 8p21 region (red) and the whole chr8p arm (green) together with 4′,6-diamidino-2-phenylindole (DAPI) (blue). Ratio of chr8p allele lengths for 5 to 10 single cells is represented as means ± SD with each dot representing one cell. Student’s *t* test was performed to determine *P* values. (**D**) Heatmap of relative gene expression of chr8 genes in chr8pWT and chr8pLOH clones determined by RNA-seq. *z*-scores for comparisons in each cell line are shown.

To further investigate the effects of potential cumulative haploinsufficiency on chr8p, we aimed to engineer liver cancer cell lines with heterozygous loss of chr8p. The TCGA-LIHC copy number data of patients with HCC revealed a region between 8p23 and 8p12 spanning 33 Mbp to be most frequently deleted leaving 9 genes before 2 Mbp and 51 genes after 35 Mbp unaffected (Fig. 2A). Therefore, we designed single guide RNAs (sgRNAs) targeting the noncoding regions at the respective boundaries of the deletion to induce double strand breaks that are subsequently repaired by the error-prone nonhomologous end-joining pathway resulting in deletion of the majority of chr8p (fig. S2A).

The three human epithelial liver cancer cell lines HLF, HLE, and HCC68 were identified to have a mainly diploid karyogram with unaltered diploid chr8p arms (Fig. 2 and fig. S2). While HLF and HLE cell lines did not show any alterations of chr8, HCC68 harbored two copies of chr8p and an amplification to three copies of chr8q (*25*). Transient introduction of the sgRNAs and subsequent single-cell sorting led to the identification of distinct single-cell clones with engineered chr8p loss (Fig. 2B). Parental wild-type (WT) single-cell clones were used to ensure a similar genetic background of WT and LOH clones. Successful deletions were determined by polymerase chain reaction (PCR) using primers flanking the deleted area and sequencing of the resulting fusion sites (fig. S2, B and C). Ultimately, we detected 21 clones with positive PCR products throughout all three cell lines, but only two HLF clones and one clone each in HLE and HCC68 cells exhibited a 50% LOH in fluorescence in situ hybridization (FISH) analysis reducing chr8p copy number from two WT alleles to one truncated and one intact chr8p (Fig. 2C). Other single-cell clones showed polyploid karyotypes with partial chr8p loss or translocation to other chromosomes. Sequencing analysis revealed nonhomologous end-joining–mediated insertions in two clones (HLF-LOH41.94 and HCC68-LOH3.230) and small indels on the remaining allele (fig. S2C). Multiplex FISH analysis and FISH staining of the whole chr8p arm and a single bacterial artificial chromosome (BAC) targeting the deleted region further confirmed LOH and excluded a possible translocation of the chr8p arm (Fig. 2C and fig. S2D). In addition, we performed whole-exome sequencing (WES) of the chr8pWT and chr8pLOH paired clones of HLF, HLE, and HCC68 cell lines to validate the heterozygous deletion of chr8p (fig. S2E). Upon chromosome engineering, cells proliferated slightly slower and exhibited morphological changes to a more spindle-like cell shape compared to their ancestral WT single-cell clones (fig. S2F). Furthermore, the engineered cell lines lost puromycin resistance, indicating that the sgRNA did not integrate (fig. S2G). Last, we performed RNA sequencing (RNA-seq) analysis of the four chr8pLOH clones together with their chr8pWT counterparts. Consistently with the human patient data (Fig. 1E), we observed a clear reduction of gene expression in the deleted area throughout all chr8pLOH clones (Fig. 2D and data S1). Together, large-scale chromosomal deletions using CRISPR-Cas9 technology were successfully introduced to establish isogenic cell clones of three different liver cancer cell lines with engineered chr8p loss.

### chr8pLOH increased metastatic capacity of cancer cells

**Fig. 3.**
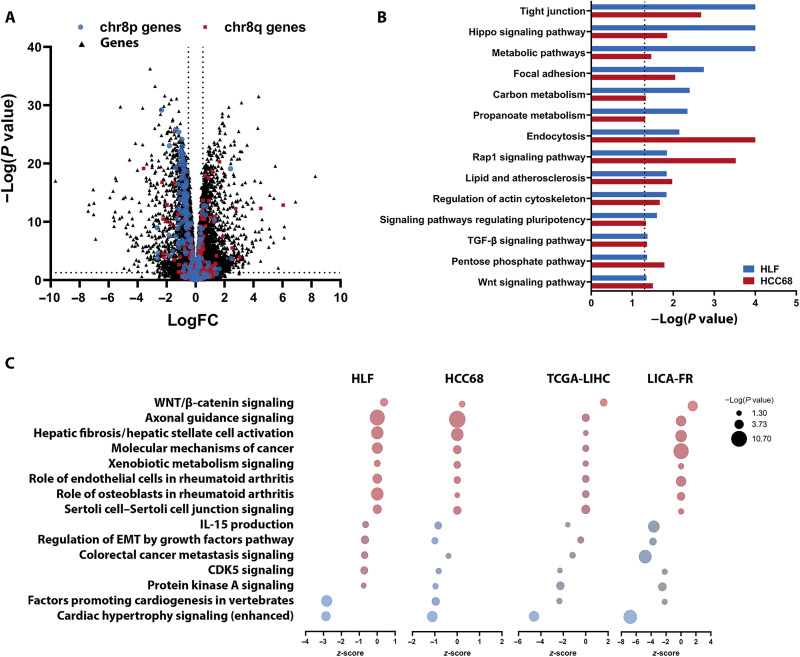
Heterozygous loss of chr8p alters genome-wide RNA expression and affects metastasis-associated pathways. (**A**) Log fold change (logFC) of genome-wide RNA expression in HLF clones determined by RNA-seq. chr8p genes are indicated in blue, and chr8q genes are indicated in red. Dotted vertical lines indicate a logFC of ±0.5 and the dotted horizontal line indicates the *P* value of 0.05 as determined by limma analysis. (**B**) Gene set enrichment analysis of chr8pLOH compared to chr8pWT clones. KEGG pathway analysis was performed for HLF and HCC68 clones individually. Pathways significantly deregulated in both cell lines are shown. (**C**) Ingenuity Pathway Analysis was performed for paired HLF clones, paired HCC68 clones, and TCGA-LIHC and LICA-FR dataset grouped by chr8pLOH and chr8pWT according to copy numbers. Only pathways significantly deregulated in all four comparisons are shown. *P* values are depicted as bubble size, and *z*-scores for pathway activation or inhibition were calculated. IL-15, interleukin-15.

Our RNA-seq data revealed that loss of chr8p did not only decrease chr8p gene expression but rather affected expression levels throughout the whole genome, including chr8q (Fig. 3A). To elucidate the corresponding signaling pathways deregulated by this large-scale perturbation, we performed gene set enrichment analysis using Kyoto Encyclopedia of Genes and Genomes (KEGG) and Ingenuity Pathway Analysis (Fig. 3, B and C, and data S2). Engineered cell clones, TCGA-LIHC, and LICA-FR expression data of patients with chr8pLOH showed common deregulated pathways including metabolic pathways and several pathways associated with cancer metastasis and cell migration [actin cytoskeleton, transforming growth factor–β (TGF-β) signaling, focal adhesion, axonal guidance, and epithelial-mesenchymal transition (EMT); Fig. 3, B and C].

**Fig. 4.**
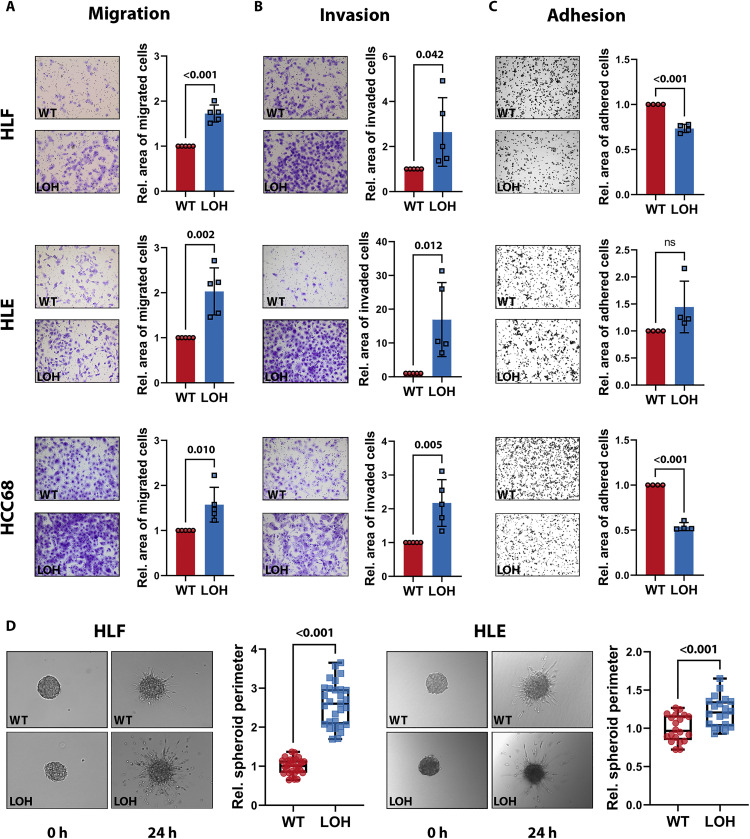
Heterozygous loss of chr8p results in a metastatic phenotype in vitro. (**A**) Transwell migration and (**B**) transwell invasion assays with quantification of migrated or invaded cell area in HLF, HLE, and HCC68 cell clones. Exemplary 10× microscopy images of transwells are shown [chr8pWT (top) and chr8pLOH (bottom)]. Data are represented as means ± SD of five independent experiments. (**C**) Cell adhesion assay and quantification of adhered cell area 1 hour after seeding in HLF, HLE, and HCC68 cell clones. Exemplary 4× microscopy images are shown [chr8pWT (top) and chr8pLOH (bottom)]. Data are represented as means ± SD of four independent experiments. (**D**) Spheroid sprouting through a collagen matrix of chr8pWT and chr8pLOH clones in HLF and HLE cells. Spheroid perimeter quantification for 20 to 30 single spheroids is represented as means ± SD of three independent experiments each dot representing one spheroid. Exemplary spheroid images are shown 0 and 24 hours after seeding. Student’s *t* test was performed to determine *P* values [*P* >0.05, not significant (ns)].

In patients with HCC or with uveal melanoma, chr8pLOH correlated with enhanced metastatic potential but direct evidence by in vitro models was missing (*19*, *26*). Copy number data of primary tumors of various entities and their respective metastases unveiled highly increased ratios of chr8p loss in the metastatic tissue compared to the corresponding primary site (fig. S3A) (*27*, *28*). Moreover, chr8p deletion was more prominent in distant than in local metastasis sites indicating chr8p loss to be a beneficial factor for the metastatic capacity of cancer cells (fig. S3B). To substantiate the effects of chr8p loss on cell motility, we analyzed the isogenic clone pairs in all three cell lines and found that chr8pLOH resulted in increased migration (Fig. 4A) and invasion (Fig. 4B). Cell adhesion was significantly decreased in HLF and HCC68 cells harboring chr8p loss (Fig. 4C), and spheroid sprouting through a collagen matrix was increased in HLF and HLE cells (Fig. 4D), while HCC68 cells did not form spheroids. All clones showed an unaltered or decreased proliferation rate, thus excluding that altered proliferation affected the observed migration phenotypes (fig. S3C).

### chr8pLOH cooperatively affected metastasis suppressors to increase migratory potential

**Fig. 5.**
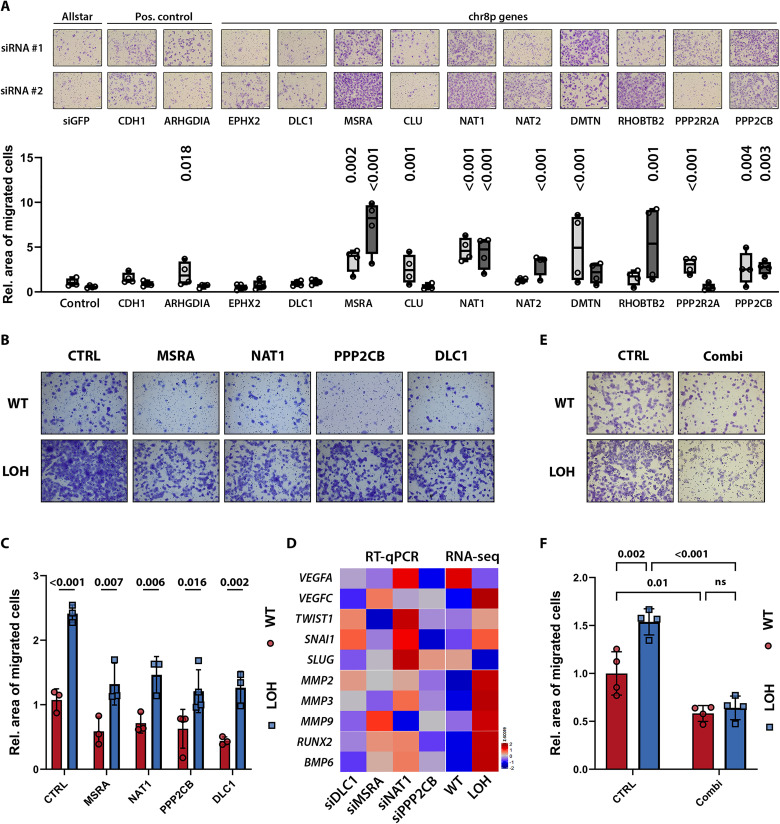
Several metastasis suppressor gene candidates are located on chr8p. (**A**) RNAi migration screen of chr8p candidate metastasis suppressors in HLF cells. Exemplary transwell migration images (top) are shown with respective quantification (bottom) of cell migration from four independent experiments. Knockdown was performed with two different siRNAs targeting each gene and quantified relative to Allstar and siGFP control (siRNA #1, light gray; siRNA #2, dark gray). Data are shown as floating bars with the line indicating median and single dots representing replicates of four independent experiments. (**B**) Representative images of transwell migration assay in chr8pWT or chr8pLOH HLF cells after transfection with empty vector (CTRL) or target gene overexpression vectors (MSRA-HA, NAT1-HA, PPP2CB-HA, DLC1-V5). (**C**) Quantification of transwell migration in chr8pWT and chr8pLOH HLF cells after gene overexpression. Data are represented as means ± SD of three independent experiments shown by single dots. (**D**) Heatmap of metastasis-associated gene expression after siRNA-mediated target gene knockdown in HLF cells compared to Allstar control [real-time quantitative PCR (RT-qPCR) data] and of chr8pWT and chr8pLOH HLF cells (RNA-seq data). *z*-scores are shown for gene expression relative to Allstar control (RT-qPCR) and relative to mean gene expression (RNA-seq). (**E**) Representative transwell migration images in chr8pWT and chr8pLOH HLF cells after transfection with empty vector (CTRL) or all four candidate genes simultaneously. (**F**) Quantification of transwell migration in chr8pWT and chr8pLOH HLF cells after gene overexpression. Data are represented as means ± SD of four independent experiments shown by single dots. Two-way analysis of variance (ANOVA) was performed for comparison of multiple groups. *P* values are indicated above the graphs (*P* > 0.05, ns).

To elucidate how chr8p loss increases metastatic potential and to identify potential metastasis suppressor genes on chr8p, we conducted RNA interference (RNAi) screening. Assuming that the expression of metastasis suppressing genes correlates with overall patient survival, we selected chr8p genes associated with significantly shortened patient survival if lowly abundant (negative Cox coefficient). We further narrowed down the selection to genes previously reported to affect cancer cell motility (fig. S4A). This resulted in 10 metastasis suppressor candidates of which methionine sulfoxide reductase A (MSRA), N-acetyltranferase 1 (NAT1), protein phosphatase 2 catalytic subunit beta (PPP2CB), and deleted in liver cancer 1 (DLC1) knockdown significantly enhanced migration with both small interfering RNA (siRNAs) tested in HLF and HCC68 cells (Fig. 5A and fig. S4B). Conversely, we tested whether overexpression of the four genes was able to hamper transwell migration of the transfected cells (fig. S4C). Congruent with our previous experiments, migration in chr8pWT HLF cells was significantly reduced following overexpression of each single gene (Fig. 5, B and C, and fig. S4, D and E). However, when overexpressed in chr8pLOH cells, neither of the chr8p metastasis suppressor candidates was able to rescue the increased migratory capacity of chr8pLOH cells completely (Fig. 5, B and C, and fig. S4, D and E). Similarly, gene expression of EMT, invasion, and metastasis markers were only partially mimicked by single-gene knockdown compared to the chr8pLOH-engineered HLF and HCC68 cells (Fig. 5D and fig. S4F). Last, the expression of all four chr8p genes, *MSRA*, *NAT1*, *PPP2CB*, and *DLC1*, together further reduced the migratory capacity of chr8pLOH cells to the level of chr8pWT cells (Fig. 5, E and F). These findings provided evidence that the action of several chr8p genes in concert accounts for the increased migration and metastasis observed in cancer cells with chr8pLOH rather than a single metastasis suppressor gene.

### Genome-wide CRISPR knockout screening revealed chr8p-dependent vulnerabilities

**Fig. 6.**
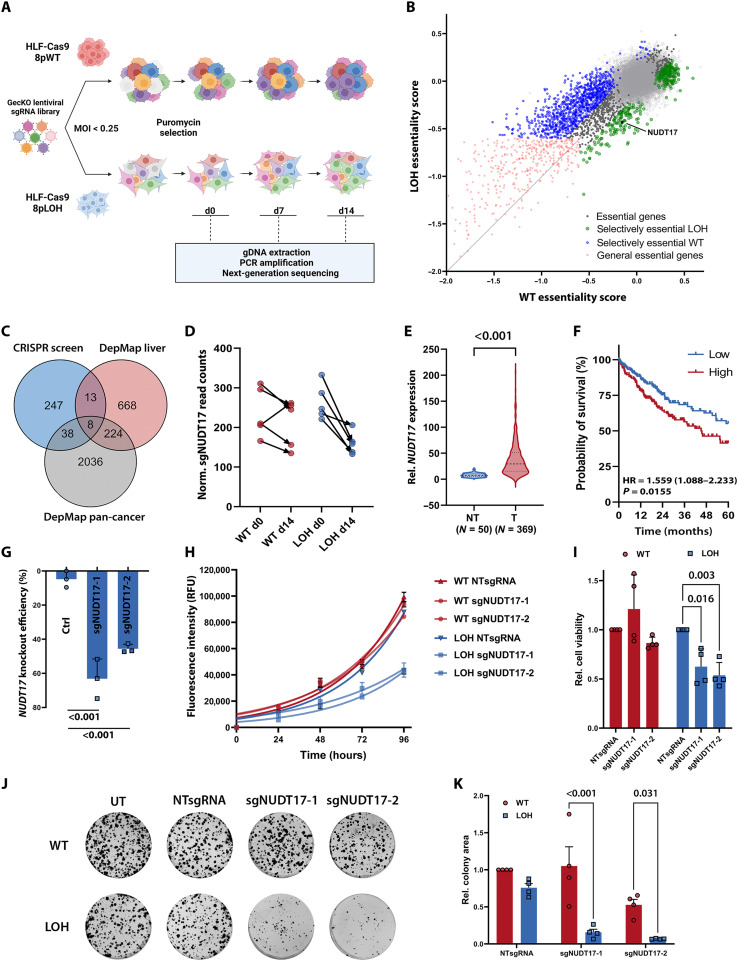
Identification of NUDT17 as chr8pLOH-specific vulnerability by genome-wide CRISPR-Cas9 knockout screen. (**A**) Schematic outline of the synthetic lethal CRISPR knockout screen performed in triplicates in chr8pWT and chr8pLOH HLF cells. (**B**) Representation of CRISPR screening results depicting essentiality scores in chr8pWT and chr8pLOH cells for each single gene. Significant (waldFDR < 0.05) genes enriched in WT cells are colored green and considered as selectively essential for chr8pLOH cells. (**C**) Venn diagram of selectively essential genes in chr8pLOH cells according to CRISPR knockout screen and DepMap analyses. (**D**) Normalized sgNUDT17 read counts in chr8pWT (red) and chr8pLOH (blue) cells at days 0 and 14. (**E**) *NUDT17* gene expression in TCGA-LIHC for normal liver (NT) and HCC (T) samples. (**F**) Kaplan-Meier survival curves of patients with TCGA-LIHC with high (red; *N* = 185) or low (blue; *N* = 184) *NUDT17* gene expression. HR with 95% confidence interval and *P* values were calculated by log-rank test. (**G**) *NUDT17* knockout efficiency of two independent sgRNAs determined by interference of CRISPR-edits (ICE) analysis in three independent experiments. (**H**) Growth curve for HLF chr8pWT and chr8pLOH cells after transduction with nontargeting sgRNA (NTsgRNA) or two independent sgRNAs targeting *NUDT17* and cell viability measurement in relative fluorescence units (RFU). Of four independent experiments, one representative growth curve is shown. Data are represented as means ± SD of technical triplicates. (**I**) Relative cell viability after 96 hours of chr8pWT and chr8pLOH cells following *NUDT17* knockout. (**J**) Representative colony formation and (**K**) quantification of colony formation area of chr8pWT and chr8pLOH HLF cells stained after 14 days. UT, untreated. Data are represented as means ± SD of four independent experiments with each dot representing the mean of one experiment. Two-way ANOVA was performed for comparison of multiple groups. *P* values are indicated above the graphs (*P* > 0.05, ns).

Pinpointing the role of large-scale genomic aberrations and the impact of corresponding signaling pathways on tumor phenotypes remains highly challenging. Therefore, it is difficult to design tailored treatment strategies specifically targeting these protumorigenic functions. Still, given the high prevalence and strong phenotype of chr8pLOH among tumor entities, the identification of vulnerabilities unique for chr8p-deleted tumors remains crucial. Taking advantage of our chromosome-engineered cell model, we performed a genome-wide CRISPR-Cas9 knockout screen to find previously unidentified chr8pLOH dependencies. Parental chr8pWT HLF and the chr8p-deleted clone HLF-LOH87 were infected with the lentiviral GeCKOv2 knockout library targeting each gene with six different sgRNAs (Fig. 6A). Next-generation sequencing was used to quantify the sgRNA abundance after 7 and 14 days and subsequently to calculate gene essentiality scores for each gene (Fig. 6A and data S3). Depletion of sgRNAs throughout the screen was represented by negative gene essentiality scores indicating a viability dependency on the gene (Fig. 6B and fig. S5A). Hereby, gene knockouts leading to reduced viability (negative gene essentiality score) in chr8pLOH but not in chr8pWT cells were of particular interest for the identification of chr8pLOH-specific vulnerabilities.

To exclude cell line–specific hits, DepMap cell lines were grouped into chr8pLOH and chr8pWT cells. This led to the identification of 914 and 2306 differential essential genes in liver cancer and pan-cancer cell lines, respectively, and these genes were overlapped with the results of our CRISPR-Cas9 screen (fig. S5, B and C). Among the eight overlapping genes, we identified *NUDT17* (Nudix-type motif 17) as one of the top candidates of chr8p-dependent vulnerabilities (Fig. 6C and fig. S5C). Targeting *NUDT17* strongly reduced the respective sgRNA abundance in chr8pLOH HLF cells over time (gene essentiality score = −0.43, Wald-test false discovery rate (waldFDR) < 0.001) but exhibited only mild effects in chr8pWT HLF cells (gene essentiality score = −0.15, waldFDR = 0.31; Fig. 6D and data S3). In addition, *NUDT17* was up-regulated in HCC tumor tissue compared to surrounding nontumor tissue (Fig. 6E) and high *NUDT17* expression correlated with decreased patient survival (Fig. 6F). Therefore, we focused our further analyses on NUDT17.

The observed NUDT17 vulnerability was validated with two independent sgRNAs exhibiting strong knockout efficiency (Fig. 6G). NUDT17 loss significantly decreased cell viability in chr8pLOH HLF cell clones, while only minor effects were detected in chr8pWT HLF cells (Fig. 6, H and I). Colony formation experiments further confirmed NUDT17 dependency, showing strongly reduced cell growth only in chr8pLOH cells lacking *NUDT17* expression (Fig. 6, J and K). Consistently, also in HCC68 and HLE cell clones, a significant synergism was observed between chr8pLOH and *NUDT17* knockout in cell viability and colony formation assays (fig. S6, A to F), confirming NUDT17 dependency in the three chr8p-deleted cancer cells HLF, HLE, and HCC68.

### NUDT17 and NUDT18 are synthetic lethal paralogs in liver cancer

**Fig. 7.**
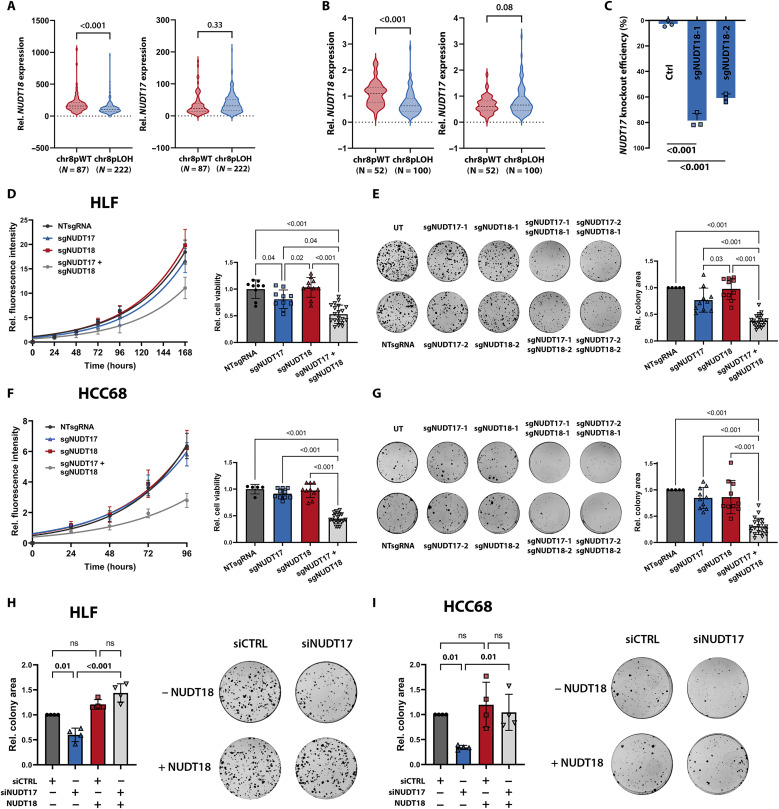
NUDT17 and the chr8p gene *NUDT18* are synthetic lethal paralogs. (**A**) *NUDT18* (left) and *NUDT17* (right) gene expression in TCGA-LIHC and (**B**) LICA-FR for chr8pWT and chr8pLOH samples. (**C**) *NUDT18* knockout efficiency of two independent sgRNAs determined by ICE analysis of three independent experiments. (**D**) Growth curves and cell viability quantification and (**E**) Colony formation assay for chr8pWT HLF or (**F** and **G**) HCC68 cells after transduction with NTsgRNA, sgNUDT17, sgNUDT18, or combinations of both (1:1 ratio). Measurements of two independent sgRNAs for single *NUDT17* or *NUDT18* knockout were combined. The combination is depicted as mean of four different sgRNA combinations. Of four independent replicates, one representative growth curve is shown with data representation as means ± SD of technical triplicates. Quantification is shown as means ± SD of four independent experiments with each dot representing one sgRNA or sgRNA combination after 96 hours (HCC68) or 168 hours (HLF). Representative colony formation images are shown 14 days after single or double knockout. Quantification data are represented as mean colony area ± SD of four independent experiments with each dot representing a sgRNA or combination of one experiment. (**H**) Colony formation rescue assays in chr8pLOH HLF or (**I**) HCC68 cells expressing *NUDT18* upon doxycycline induction and transfected with siPools targeting *NUDT17*. Quantification is shown as mean colony area ± SD of four independent experiments with each dot representing a single experiment 14 days after seeding. Representative images of four replicates are shown. Two-way ANOVA was performed for comparison of multiple groups. *P* values are indicated above the graphs (*P* > 0.05, ns).

We then elucidated the nature of the observed NUDT17 dependency in liver cancer cell lines. The more thoroughly studied Nudix family gene *NUDT18* is considered a paralog to *NUDT17* and is located on chr8p21.3. For multiple NUDT family members, including NUDT18, roles in hydrolyzing oxidized nucleotides initiating their degradation have been proposed (*29*). Consistently with its location on chr8p, *NUDT18* expression was down-regulated in chr8pLOH patients of the TCGA-LIHC and LICA-FR datasets (Fig. 7, A and B). In addition, *NUDT18* expression was reduced in the engineered chr8pLOH cell lines compared to the chr8pWT cell lines (fig. S7A). *NUDT17* expression, on the other hand, was not significantly affected upon chr8p loss in the TCGA-LIHC dataset (Fig. 7A), the LICA-FR dataset (Fig. 7B) nor the engineered chr8pLOH cell lines (fig. S7A). Consistently, *NUDT18* was in contrast to *NUDT17* not up-regulated in tumor compared to nontumor tissue samples (fig. S7B) and not associated with patient survival (Fig. 6F and fig. S7C).

We hypothesized that NUDT17 and NUDT18 are synthetic lethal paralogs explaining the observed NUDT17 dependency in chr8pLOH cells. To test this hypothesis, we performed cell viability and colony formation assays in chr8pWT HLF and HCC68 cells after knockout of *NUDT17* and *NUDT18* alone or in combination. Therefore, two sgRNAs exhibiting strong knockout efficiency (Figs. 6G and 7C) against each gene were used, either alone or in four different combinations to target both genes simultaneously. Whereas *NUDT17* knockout alone resulted only in minor antiproliferative effects in HLF cells, the combined knockdown of *NUDT17* and *NUDT18* showed a strong reduction of cell viability and colony formation in both cell lines suggesting therapeutic exploitability (Fig. 7, D to G). To independently confirm the knockout experiments, we validated the effects of NUDT17 loss on chr8pLOH cells also in an orthogonal approach using RNAi pools with 30 different siRNAs targeting *NUDT17* (fig. S7D). In addition, we were able to show that ectopic *NUDT18* overexpression rescued the effects of NUDT17 knockdown in chr8pLOH HLF and HCC68 cell clones substantiating the synthetic lethal relation of the two paralogs (Fig. 7, H and I, and fig. S7E). Notably, both overexpression and knockdown of NUDT17 or NUDT18 did not affect the expression of the respective paralog (fig. S7F). This confirmed that the NUDT17 dependency in chr8p-deleted tumor cells is caused by reduced *NUDT18* expression. Hence, we propose the existence of a unique vulnerability of liver cancer cells harboring a chr8p-deleted background due to their NUDT17 dependency.

**Fig. 8.**
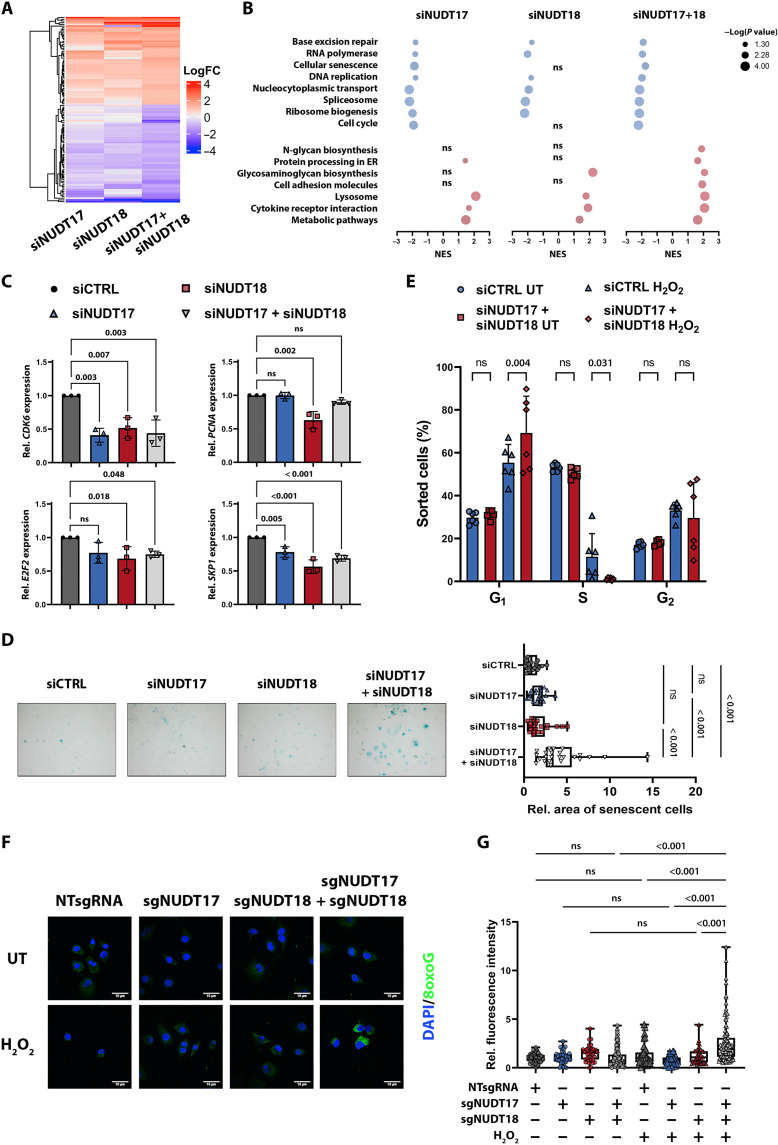
Concomitant NUDT17/18 loss leads to cell cycle arrest. (**A**) Heatmap depicting log fold gene expression of the most prominently altered genes after pooled siRNA-mediated knockdown of NUDT17 and NUDT18 as determined by RNA-seq in HLF cells. (**B**) KEGG gene set enrichment analysis of NUDT17, NUDT18, or combinatory knockdowns relative to control transfected HLF cells. Pathways significantly deregulated after double knockdown are shown. ER, endoplasmic reticulum. (**C**) RT-qPCR validation of cell cycle–associated gene expression in HCC68 cells. (**D**) β-Galactosidase staining of HLF cells after knockdown of NUDT17 and NUDT18. Exemplary images with senescent cells visualized in blue. Quantification of relative stained area is depicted as box whisker plots with each dot representing one image of three independent experiments (**E**) Cell distribution in G_1_, S, and G_2_ cell cycle phases after dual knockdown untreated (UT) or treated with 5 μM H_2_O_2_ was analyzed by flow cytometric measurements of EdU-incorporating proliferative cells and FxCycle-FarRed DNA staining. (**F**) Immunofluorescence images of HLF cells after single or dual knockout and treatment with 10 μM H_2_O_2_. Nuclei were stained with DAPI in blue and cytosolic 8oxo–2′-deoxyguanosine 5′-triphosphate (dGTP) levels are shown in green. Knockout was performed with two independent sgRNAs for both genes. Exemplary images are shown for each condition. (**G**) Quantification of relative intensity of 8oxo-dGTP immunofluorescence. Data are represented as box whisker plots with each dot representing one single cell of three to six independent experiments. Image analysis was performed using Fiji software. Two-way ANOVA was performed for comparison of multiple groups. *P* values are indicated above the graphs (*P* >0.05, ns).

To gain mechanistic insight in the rather enigmatic function of NUDT17, we next performed RNA-seq in HLF cells after single or double knockdown of NUDT17 and NUDT18 (Fig. 8A and data S1). We detected a similar expression patterns for both gene knockdowns, which was further intensified by a combined inhibition. Gene set enrichment analysis revealed down-regulation of DNA repair pathways, cell cycle progression, and senescence in HLF cells (Fig. 8B and data S2). Deregulation of respective cell cycle (*CDK6*, *PCNA*, *E2F2*, and *SKP1*), DNA repair (*RPA1*, *RPA2*, and *TDG*), and senescence-related genes (*CXCL2* and *CXCL3*) was independently confirmed in HCC68 cells (Fig. 8C and fig. S8A). Effects on cell senescence were further confirmed by increased β-galactosidase staining of cells lacking expression of both proteins, NUDT17 and NUDT18 (Fig. 8D). Consistently, previous reports have reinforced our findings by suggesting a role for NUDT18 in the response to reactive oxygen species (ROS) by clearing ROS damage products and reducing subsequent DNA damage (*29*, *30*). Thus, we hypothesized that NUDT17 and NUDT18 have overlapping functions and are essential for clearing ROS damage products subsequently reducing DNA damage. Upon ROS induction with H_2_O_2_, cells lacking NUDT17 and NUDT18 protein expression showed G_1_ arrest and reduction of proliferative S phase cells compared to control cells transfected with siCTRL (Fig. 8E and fig. S8, B and C). Besides, a strong cytosolic accumulation of the mutagenic oxidative damage product 8oxoguanine (8oxoG) was observed upon NUDT17 and NUDT18 loss together with increased oxidative stress in both HLF and HCC68 cell lines, whereas deletion of NUDT17 or NUDT18 individually did not have any significant effect (Fig. 8, F and G, and fig. S8, D and E). This suggested a common role of NUDT17 and NUDT18 in the clearance of 8oxoG upon oxidative stress resulting in the inability to remove DNA damaging ROS products. In conclusion, we propose that NUDT18 reduction by chr8pLOH sensitizes these tumor cells for NUDT17 targeting. This represents a unique dependency solely of cancer cells harboring a chr8p-deletional status that might be exploited therapeutically in the future.

## DISCUSSION

Although copy number changes and large chromosomal alterations are highly prevalent throughout most cancer types, it has been technically challenging to model large chromosomal alterations in human cells. The emergence of a continuously refined toolbox of genome-editing technologies made it possible to alter large chromosomal regions at the megabase scale (*2*, *17*). Cai *et al.* (*17*) used transcription activator-like effector nucleases (TALEN) to delete chr8p in a single breast cell line that caused metabolic alterations. Here, we established three human HCC models using CRISPR-Cas9 technology to study the effect of heterozygous loss of chr8p, frequently observed in human carcinogenesis. Using this clinically relevant cellular model, we not only elucidated the role of metastasis suppressing genes on chr8p but also exploited chr8p-dependent vulnerabilities.

Mimicking the deletion most frequently found in patients with HCC, we observed a profound increase of metastatic potential in chr8pLOH cells compared to their isogenic chr8pWT counterparts. This is in line with a previous study indirectly correlating increased cancer metastasis with chr8p loss in colorectal cancer (*31*). Xue *et al.* (*16*) performed an in vivo screen including genes in the genomic region of *DLC1* on chr8p that led to the identification of genes increasing the tumorigenic potential in a p53/Myc HCC model. We focused our siRNA screen on cell migration and thereby confirmed a metastasis suppressive role for MSRA, NAT1, PPP2CB, and DLC1 in liver cancer. Among those, DLC1 has been widely acknowledged as an effector of metastatic potential (*32*–*34*). However, in our studies DLC1 reconstitution failed to completely rescue the effects of chr8pLOH on cancer cell migration, similar to the other metastasis suppressor candidates indicating cooperating roles of these genes in suppressing invasiveness. Consistently with these results, it has been suggested that for large-scale deletions, the concomitant deregulation of many genes rather than single perturbations may drive cancerous traits (*16*, *17*). This deregulation is highly cell type and context dependent, allowing for a broad transcriptional heterogeneity and providing the tumor with selective advantages with regard to therapy resistance and metastasis. It should be noted that although a strong induction of migration and invasiveness has been observed in chr8pLOH cells, this only yields information for an EMT phenotype and dissemination from the primary tumor. Investigation of colonization at distant sites, however, is much more intricate. Circulating cells need to acquire opposing mesenchymal-epithelial transition–like features to extravasate into the metastatic site and need to adapt to the new environment, thereby requiring a turnover transcriptional reprogramming (*35*).

With regard to systemic therapeutic approaches, a fine balance has to be kept between targeting the aggressive tumor and preserving essential liver functions. This is of particular importance for liver cancer patients because most HCC arise in the background of chronic liver disease and have already compromised liver capacity (*36*). Systemic therapy for HCC currently includes the multikinase inhibitors sorafenib or lenvatinib and immunotherapy-based combinations; however, only a subset of patients benefits from those therapeutics due to rapid resistance or toxicity (*36*). To minimize adverse effects, synthetic lethality strategies have great advantages especially in targeting DNA damage repair pathways, exploiting passenger vulnerabilities of cancer cells that can be compensated by nonmalignant cells (*37*–*39*). Thus, large-scale vulnerability screens have been a powerful tool to identify synthetic lethalities (*40*). Our unique HCC model of chr8pLOH allowed the implementation of such screening approaches to untangling patient-specific vulnerabilities.

**Fig. 9.**
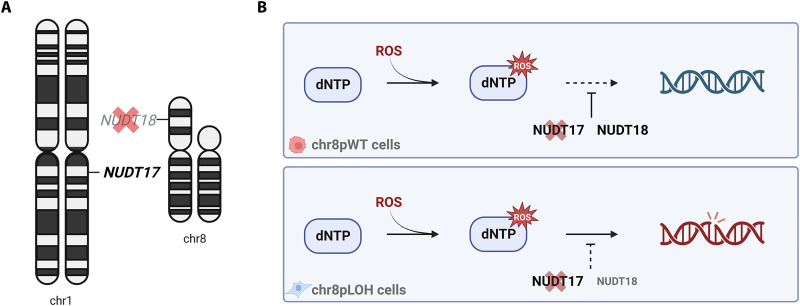
NUDT17 and NUDT18 are synthetic lethal paralogs affecting ROS response. (**A**) Illustration on NUDT17 dependency in cells harboring chr8pLOH. Loss of NUDT18 in chr8pLOH increases dependency on its paralog NUDT17. (**B**) Reduced levels of NUDT18 in cells with chr8pLOH create NUDT17 dependency. Upon NUDT17 ablation, chr8pLOH cells are prone to DNA damage by ROS leading to impaired cell viability and cell cycle arrest, whereas chr8pWT cells remain unaffected.

We showed that the NUDT17 is a candidate target allowing to selectively inhibit chr8p-deleted tumor cells. We performed in-depth validation of our CRISPR-Cas9 screen in independent isogenic cell clones of three different cell lines that clearly validated that lack of the NUDT18 paralog in chr8pLOH tumor cells sensitized them to NUDT17 ablation. NUDT17 and NUDT18 have high structural similarities and belong to a family of 22 human hydrolases characterized by a shared Nudix domain (*29*, *41*). NUDT family members have been suggested to share ambiguous roles in the hydrolysis of phosphorylated nucleotides involving physiological and therapeutic metabolites (*41*, *42*). However, the roles of distinct NUDT family members remain enigmatic and demand further elucidation, particularly in unraveling their substrate specificity (*43*). Our results suggested a shared function of NUDT17 and NUDT18 in clearance of oxidative damage. Given the NUDT18 deficiency in chr8pLOH cells, targeting NUDT17 exerted a strong effect only on these cells but did not affect cells with unaltered chr8p status (Fig. 9A). We showed that cells lacking both NUDT family members are prone to DNA damage by ROS leading to impaired cell viability and cell cycle arrest (Fig. 9B). Notably, NUDT1 and NUDT15 have been closely associated with NUDT17 and NUDT18 in phylogenetic and structural studies suggesting functional similarities (*29*, *30*, *41*). However, only NUDT1 has been in the focus for the development of selective inhibitors, and the other family members have not been chemically targeted so far (*44*, *45*). Similar to NUDT18, NUDT1 is a major cleanser of the cytosolic pool of oxidized nucleotides and has been studied as a potential target in several cancer entities (*45*–*48*). Many tumors show increased levels of ROS that can be even beneficial for tumor progression. Increasing the amount of ROS and hampering its clearance in cancer cells by inhibition of Nudix hydrolases induce cell death if the cells are unable to repair the accompanied damage (*49*, *50*). In an organ continuously exposed to toxic agents, such as the liver, targeting ROS response is a promising strategy, as NUDT17 addiction is specific to chr8p-deleted cancer cells.

In recent years, a growing number of synthetic lethal gene pairs have been proposed (*51*, *52*). While the field of DNA damage response has been a leading example for the efficacy of the strategy, it has been translated to many different pathways and mechanisms (*53*). Although targeting cancer drivers has been successfully applied in several tumor entities, in many others, including HCC, chemotherapeutic and immunotherapeutic approaches showed limited effectiveness. With the advent of precision therapeutics and promising concepts aiming to allow drugging of virtually any protein (*54*, *55*), powerful models to connect tumor-specific alterations with potential synthetic lethal targets are promising. Large-scale chromosomal deletions are highly prevalent throughout many human cancers and can be routinely detected in cancer diagnostics, but only recently models to study them in detail have been described (*56*, *57*). Our model permitted in-detail characterization of tumor biology in chr8p-deleted tumors leading to a better understanding of the mechanisms driving increased tumor progression and metastasis. Furthermore, we showed how this model can be used to predict patient-specific synthetic lethality.

Although we believe that chromosome-engineered cell lines represent a powerful tool, some limitations remain. On the one hand, extravasation properties can hardly be investigated with in vitro models only. To investigate how cells can enter distant tissue environment from the vascularization system, a lung metastasis model in immune-deficient mice could be applied. However, this technique is strongly cell line dependent, and we tested our HLF and HCC68 cell lines in different immune-deficient mouse strains, but neither chr8pWT nor chr8pLOH cells led to successful lung colonization. A second limitation is caused by the unique chromosomal landscape of humans, which often does not resemble the mouse chromosome architecture. This makes it impossible to investigate chr8p loss in murine cells.

Still, this study serves as a proof of concept to advance chromosome engineering to other human cancers or frequent copy number alterations and thereby supplementing drug discovery with a valuable tool to link copy number alterations with potential tumor vulnerabilities. We successfully performed a genome-wide CRISPR-Cas9 viability screening in isogenic chr8p-deleted cells and thereby found previously unidentified synthetic lethal targets and vulnerabilities accompanying chr8p deletion. Using this target identification strategy, we found that chr8p deletion sensitizes tumor cells to targeting of the reactive oxygen sanitizing enzyme NUDT17. The development of NUDT17 specific inhibitors will allow for specific targeting of tumor cells with chr8p deletion without affecting normal cells. Thus, chromosomal engineering led to the identification of previously unidentified synthetic lethalities specific to chr8pLOH.

## MATERIALS AND METHODS

### Experimental design

The purpose of this study was to investigate how heterozygous loss of chr8p affects cancer progression and to identify potential targets specific for patients harboring this chromosomal alteration. Using a chromosome-engineering strategy, an in vitro platform of human liver cancer was established mimicking chr8p deletion. Given its exclusivity in humans, a holistic study of chr8p deletion could not be performed in mice. chr8p loss was validated in-depth by sequencing, FISH and expression analysis, effects on tumor biology were tested by intensive functional characterization. To identify specific targets a CRISPR-Cas9 viability screening was performed in chromosome-engineered cell lines and integrated with essentiality data of the DepMap database to exclude cell line–specific effects. The in vitro platform served as tool to validate targets and get mechanistic insights.

### Cell culture

The liver cancer cell lines HLF, HLE, HCC68, and the human embryonic kidney (HEK) 293T cells were used in this study. Cell lines were obtained from American Type Culture Collection (HEK293T) or Japanese Collection of Research Bioresources (HLF and HLE). HCC68 cells were provided by J. Marquardt. Cells were regularly tested for mycoplasma contamination (MycoAlert, Lonza, Basel, Switzerland) and authenticated by short tandem repeat (STR) analysis. All cells were cultured in Dulbecco’s modified Eagle’s medium (DMEM; Sigma-Aldrich, Taufkirchen, Germany). The growth medium was supplemented with 10% fetal bovine serum (FBS) and 1% penicillin/streptomycin (both Thermo Fisher Scientific, Waltham, MA, USA).

### Transfection of cells

For siRNA-mediated knockdown, Lipofectamine RNAiMAX Transfection Reagent (Thermo Fisher Scientific, Waltham, MA, USA) was used according to the manufacturer’s instructions. siRNAs were obtained from QIAGEN and are listed in data S4 (QIAGEN, Germantown, MD, USA), siPools were obtained from siTools and were used at 1 nM concentration according to the manufacturer’s protocol (siTOOLs Biotech, Martinsried, Germany).

Transient expression of sgRNAs and target gene cDNAs was achieved by transfecting modified pX458 vector (pX458-tRNA-sg2MB-tRNA-sg35MB-dCas9-Puro) and pDEST or pcDNA plasmids (pDest-MSRA-HA, pDest-NAT1-HA, pDest-PPP2CB-HA, pDest-Ctrl, and pcDNA-DLC1), respectively, using FuGene transfection reagent (Promega, Walldorf, Germany) in a 1:3.5 ratio according to the manufacturer’s instructions.

### Generation of stable expression cell lines

For generation of lentiviral particles, HEK293T cells were transfected with a transfection mix containing 1 ml of optimized minimal essential medium (Thermo Fisher Scientific, Waltham, MA, USA), 60 μl of PEI (Polysciences, Warrington, PA, USA), packaging vectors pMD2.G (2.5 μg; Addgene #12259) and psPAX2 (8 μg; Addgene #12260), and 10 μg of expression vector (lentiCas9-Blast, Addgene #52962) (*58*). Growth medium was changed after 16 hours, and viral particles were collected after additional 24 hours by filtering supernatant using 0.45-μm Millex-HA filter (Merck Millipore, Burlington, MA, USA). HLF, HLE, and HCC68 cell lines were infected with viral particles and selected with blasticidin (10 μg/ml; Thermo Fisher Scientific, Waltham, MA, USA) for 2 weeks.

### Establishment of chr8pLOH cells

For chromosome engineering, sgRNAs were designed using chopchop.cbu.uib.no website targeting noncoding regions at 2 and 35 Mbp of chr8p. Constructs expressing both sgRNAs were introduced transiently into Cas9-expressing cells (HLF-Cas9, HLE-Cas9, and HCC68-Cas9) as described above. Cells were selected for 2 days by puromycin supplementation (1 μg/ml; Thermo Fisher Scientific, Waltham, MA, USA), and subsequently single cells were sorted into 96-wells using a BD FACSAria III cell sorter. Single-cell clones were expanded incrementally to six-well plates, and genomic DNA (gDNA) was isolated using the QIAGEN Gentra Puregene Kit (QIAGEN, Germantown, MD, USA). Single-cell clones were screened for chr8p deletion by PCR using primer pairs flanking the deleted region (data S4). Positive clones were Sanger-sequenced (Mycrosynth SeqLab, Göttingen, Germany), and LOH was confirmed by FISH analysis.

### Crystal violet kill assay

To exclude genomic integration of the pX458-tRNA-2 Mbp-tRNA-35 Mbp-dCas9-Puro vector in the chr8pLOH clones and to validate the stable integration of the Cas9-blasticidin cassette into single-cell clones, 10,000 cells were seeded into 24-well plates and treated with puromycin (2 μg/ml) and blasticidin (10 μg/ml) or left untreated. Cells were cultured for 7 to 14 days, washed with phosphate-buffered saline (PBS), and stained with 0.5% crystal violet solution (Sigma-Aldrich, Taufkirchen, Germany) in 25% methanol (Honeywell, Charlotte, NC, USA) for 30 min at room temperature.

### Fluorescence in situ hybridization

Cells were treated with colcemid (0.135 μg/ml; Thermo Fisher Scientific, Waltham, MA, USA) and incubated for 2 to 4 hours at 37°C in a 5% CO_2_ incubator. Subsequently, cells were trypsinized and underwent hypotonic treatment (0.55% KCl and 1% Na citrate, 2:1 volume ratio) for 20 min at 37°C. Cells were fixed by slowly adding methanol and glacial acid (3:1 volume ratio).

Two-color FISH experiments were performed using the following BAC clones for chromosome regions: 8p23.3 [BAC114J18 (hg19): 980,591 to 1,141,036 bp], 8p21.3 [RP11-177H13 (hg19): 23,051,365 to 23,230,686 bp], 8p21.2 [RP11-14I17 (hg19): 26,150,540 to 26,320,613 bp], and 8p12 [RP11-197P20 (hg19): 37,085,323 to 37,252,083 bp), respectively, together with a partial painting probe for the short arm of chr8 (pcp8p). FISH results were evaluated using a DM RXA epifluorescence microscope (Leica Microsystems, Bensheim, Germany) equipped with a Sensys charge-coupled device camera (Photometrics, Tucson, AZ). Camera and microscope were controlled by the Leica Q-FISH software.

### Multiplex fluorescence in situ hybridization

Multiplex FISH was performed as described by Geigl *et al.* (*59*). Briefly, seven pools of flow-sorted whole chromosome painting probes were amplified and combinatorial labeled using diethylaminocoumarin-, fluorescein isothiocyanate–, Cy3-, TexasRed-, and Cy5-conjugated nucleotides and biotin–deoxyuridine triphosphate and digoxigenin–deoxyuridine triphosphate, respectively, by degenerative oligonucleotide primed–PCR. Prior hybridization, metaphase spreads fixed on glass slides were digested with pepsin (0.5 mg/ml; Sigma-Aldrich, Taufkirchen, Germany) in 0.2 N HCL (Roth, Karlsruhe, Germany) for 10 min at 37°C, washed in PBS, postfixed in 1% formaldehyde, dehydrated with a degraded ethanol series, and air-dried. Slides were denaturated in 70% formamide/1× saline-sodium citrate (SSC) for 2 min at 72°C. Hybridization mixture containing combinatorial labeled painting probes, an excess of unlabeled cot1 DNA in 50% formamide, 2× SSC, and 15% dextran sulfate were denaturated for 7 min at 75°C, preannealed for 20 min at 37°C, and hybridized to the denaturated metaphase preparations. After 48 hours of incubation at 37°C, slides were washed at room temperature in 2× SSC for 3× 5 min, followed by 0.2× SSC/0.2% Tween 20 at 56°C for 2× 7 min. For indirect labeled probes, an immunofluorescence detection was carried out. Therefore, biotinylated probes were visualized using three layers of antibodies: streptavidin Alexa Fluor 750 conjugate (Thermo Fisher Scientific, Waltham, MA, USA) and biotinylated goat anti avidin (Vector Laboratories, Newark, CA, USA), followed by a second streptavidin Alexa Fluor 750 conjugate (Thermo Fisher Scientific, Waltham, MA, USA). Digoxigenin-labeled probes were visualized using two layers of antibodies: rabbit antidigoxin (Sigma-Aldrich, Taufkirchen, Germany), followed by goat anti rabbit IgG Cy5.5 (Linaris, Germany). Slides were washed in between in 4× SSC/0.2% Tween 20 for 3× 5 min, counterstained with 4′,6-diamidino-2-phenylindole (DAPI), and covered with antifade solution. Images of metaphase spreads were captured for each fluorochrome using highly specific filter sets (Chroma Technology, Brattleboro, VT) recorded using a DM RXA epifluorescence microscope (Leica Microsystems, Bensheim, Germany) equipped with a Sensys charge-coupled device camera (Photometrics, Tucson, AZ). Camera and microscope were controlled by the Leica Q-FISH software, and images were processed on the basis of the Leica MCK software and presented as multicolor karyograms (Leica Microsystems Imaging solutions, Cambridge, United Kingdom).

### Whole-exome sequencing

gDNA of chr8pWT and chr8pLOH cell clones was isolated using the QIAGEN Gentra Puregene Kit (QIAGEN, Germantown, MD, USA). For WES, the Twist Human Core Exome, Twist Human RefSeq, and Twist Mitochondrial panels (Twist Bioscience, San Francisco, CA, USA) panels were used for library preparation. Sequencing was performed on the NovaSeq 6000 (Illumina, San Diego, CA, USA) with 2× 101 bp.

WES samples in FASTQ format were analyzed using the nf-core/sarek v3.2.3 pipeline (*60*, *61*). Quality control and trimming were performed using FASTQC (www.bioinformatics.babraham.ac.uk/projects/fastqc/) and Fastp (*62*). Reads were aligned to the human reference genome GRCh38 using BWA (*63*), and duplicates were marked using GATK MarkDuplicates (*64*). Variant calling was performed using mutect2 and variant annotation using snpEff (*65*). To assess driver mutations, we annotated the detected variants of each cell line using the cancer genome interpreter (*66*). Copy number variations were analyzed and plotted by CNVkit using paired samples between chr8pWT and chr8pLOH (*67*).

The raw data are deposited in the Sequence Read Archive (www.ncbi.nlm.nih.gov/sra/; project ID PRJNA994866; run IDs SRR25278406 to SRR25278417).

### RNA sequencing

RNA-seq was performed for chr8pWT and chr8pLOH cell clones and for HLF cells with NUDT17 and/or NUDT18 inhibition. Total RNA was extracted using the QIAGEN RNAeasy kit, and, subsequently, 2 μg of total RNA was sent to BGI Hong Kong Tech Solution for mRNA and long noncoding RNA-seq using the DNBseq platform. For library preparation, ribosomal RNA was depleted from the samples, and cDNA was synthesized with respective adaptor sequences. Sequencing was performed with phi29, and single-end 50-base reads were generated in the way of combinatorial probe-anchor synthesis.

Analysis of RNA-seq data was done with R and Bioconductor using the next-generation sequencing analysis package systempipeR (*68*). Quality control of raw sequencing reads was performed using FastQC (www.bioinformatics.babraham.ac.uk/projects/fastqc/). Low-quality reads were removed using trim:galore (version 0.6.4). The resulting reads were aligned to human genome version GRCh38.p13 from GeneCode and counted using kallisto version 0.46.1 (*69*). The count data were transformed to log_2_ counts per million, estimated the mean-variance relationship, and used this to compute appropriate observational level weights for linear modeling using the voom function from the limma package (*70*). Differential expression analysis was performed using the limma package in R. A false-positive rate of α = 0.05 with FDR correction was taken as the level of significance. The raw and normalized data were deposited in the Gene Expression Omnibus database (accession number GSE220320).

Gene set enrichment analysis was performed using either the KEGG database or QIAGEN Ingenuity Pathway Analysis software. KEGG pathway analysis was made with fgsea package (*71*, *72*) and the enrichmentbrowser package (*73*) in R using the pathway information from KEGG database (www.genome.jp/kegg/pathway.html). Analyzed RNA-seq data and enriched pathways are shown in data S1 and S2.

### Real-time quantitative PCR

Gene expression analysis by real-time quantitative PCR (RT-qPCR) was performed by isolating RNA from liver cells using NucleoSpin RNA Kit (Machery-Nagel, Düren, Germany) as stated in the manufacturer’s protocol. Five hundred nanograms of total RNA was transcribed to cDNA following the Takara PrimeScript RT Reagent protocol (Takara Bio Europe SAS, Saint-Germain-en-Laye, France). Amount of cDNA was measured on a StepOnePlus real-time PCR system (Applied Biosystems, Darmstadt, Germany) using primaQuant CYBR-Green Mastermix low ROX (Steinbrenner Laborsystems, Wiesenbach, Germany). Relative mRNA expression was calculated using the comparative *C*_t_ method with the reference gene serine- and arginine-rich splicing factor 4 for normalization. Primers were obtained from Thermo Fisher Scientific (data S4).

### Protein isolation and Western blot

Protein was isolated from human liver cancer cell lines using cell lysis buffer 10× (Cell Signaling Technology) supplemented with protease inhibitor and phosphatase inhibitor PhosStop (Roche Diagnostics, Mannheim, Germany). Cell lysis was enhanced by 30-s sample sonification. Protein concentrations were measured by Bradford assay (Sigma-Aldrich, Taufkirchen, Germany) and diluted to final concentration of 1 μg/ml in 1× Laemmli sample buffer [250 mM tris-HCl (pH 6.8), 8% SDS, 40% glycerol, 100 mM dithiothreitol, and 0.04% bromophenol blue]. Of the protein samples, 30 μg were separated on 8 to 12% bis/tris-polyacrylamide gels and then transferred to nitrocellulose membranes (Merck Chemicals, Darmstadt, Germany). Membranes were blocked with 5% milk in tris-buffered saline with Tween 20 (TBST) or 5% bovine serum albumin in TBST and incubated with the primary antibodies overnight at 4°C (data S4). Proteins were detected with IRDye secondary antibodies using an Odyssey Sa Infrared Imaging System (LI-COR Biosciences, Bad Homburg, Germany).

### Transwell migration and invasion assays

Cells were transfected or treated and cultured in medium containing 5% FBS for 16 hours before starving for 4 hours in serum-free medium. Starved cells were detached using Trypsin-EDTA (Sigma-Aldrich, Taufkirchen, Germany), and the reaction was stopped by adding defined trypsin inhibitor (Thermo Fisher Scientific, Waltham, MA, USA) and resuspended in serum-free medium. Cells were counted, and 50,000 cells in 500 μl were seeded either in Falcon Permeable Support (8.0 μm) or in previously equilibrated BioCoat Matrigel Invasion Chambers (8.0 μm) (both Corning Incorporated, NY, USA) for migration and invasion assay, respectively. Transwells were placed into Falcon 24-well companion plate (both Corning Incorporated, NY, USA) containing 800 μl of medium supplemented with 10% FBS. Receiver wells with serum-free medium were used as negative control and normalization blank. Cells were incubated in transwells for 24 hours at 37°C. Cells inside the wells were removed and migrated or invaded cells on the apical site that were stained using crystal violet (Sigma-Aldrich, Taufkirchen, Germany). Transwells were microscopically imaged, and the area of migrated or invaded cells was quantified using Fiji software to measure migratory or invasive capacity.

### Adhesion assay

Cells were treated or left untreated, and 100,000 cells were seeded on 12-well plates. Growth medium was removed 1 hour after seeding, washed twice with PBS, and stained using crystal violet (Sigma-Aldrich, Taufkirchen, Germany). Attached cells were quantified using Fiji software after microscopical image acquisition using an Olympus CKX41 microscope with an XM10 camera at ×4 magnification using the Olympus CellSens Dimension software.

### Three-dimensional spheroid sprouting assay

A hanging drop approach was used to generate spheroids. Cells were trypsinized after respective treatment, and 100,000 cells were resuspended in medium containing 20% (v/v) METHOCEL solution (12 mg/ml; Sigma-Aldrich, Taufkirchen, Germany). Of the cell suspension, 20 μl were seeded as hanging drops on the lids of a 15-cm dish, inverted, and incubated at 37°C in a 5% CO_2_ incubator for 48 hours. Coating of 12-well plates was performed with 250 μl of Collagen:METHOCEL solution [containing 10× DMEM (Sigma-Aldrich, Taufkirchen, Germany), METHOCEL, and Purecol (Advanced Biomatrix, Carlsbad, CA, USA)]. Spheroids were carefully resuspended in medium and pelleted, and 20 to 30 spheroids were seeded in additional 250 μl of Collagen:METHOCEL solution on top of the precoated wells. After polymerization, 500 μl of medium supplemented with 10% FBS was added on top of the Collagen matrix. Image acquisition was performed at indicated time points using an Olympus CKX41 microscope with a XM10 camera at ×4 and ×10 magnifications using the Olympus CellSens Dimension software. Invasion capacity was quantified by measuring the perimeter of the spheroids and corresponding sprouts using the Fiji software. All perimeters were normalized to the mean perimeter of the WT control spheroids at day 0.

### 5-Bromo-2′-deoxyuridine proliferation assay

Cell proliferation was analyzed using a 5-bromo-2′-deoxyuridine (BrdU)–enzyme-linked immunosorbent assay (ELISA) approach according to the Amersham Cell Proliferation ELISA Biotrak System (GE Healthcare, Chicago, IL, USA). Therefore, 6000 cells were seeded in 96-well plates and incubated with BrdU reagent for 120 min at 37°C. After BrdU incorporation, cells were fixed and subsequently blocked for 30 min. BrdU antibody (50 μl) was added to each well (1:250 dilution) for 120 min. After washing, 50 μl of trimethylboron substrate was added for 5 min before termination of the reaction by addition of 12.5 μl of 1 M sulfuric acid. Absorbance was measured at 450 nm with an Omega FLUOstar Microplate Reader (BMG LABTECH, Ortenberg, Germany).

### RNAi transwell migration screening

Candidate chr8p genes were selected by analyzing TCGA survival datasets. List of genes with significant negative cox coefficient was further narrowed down by literature research for association with migration and metastasis. Each gene was knocked down individually by two predesigned QIAGEN FlexiPlate siRNAs, and knockdown efficiency was validated by RT-qPCR (data S4). In addition, two negative controls (Allstars and siGFP) and positive controls (siCDH1 and siARHGDIA) were used. For reverse transfection, 200,000 HLF or HCC68 cells were incubated with 10 μM of each siRNA for 48 hours. Cells were starved for 4 hours, then seeded in in FBS-free growth medium in Falcon Permeable Support 8.0-μm transwells, and incubated for 20 hours at 37°C. Apical transwells were stained with crystal violet (Sigma-Aldrich, Taufkirchen, Germany) and analyzed using the Fiji software as described above. Area of migrated cells was normalized to the mean area of the negative controls.

### CRISPR-Cas9 knockout screening

The genome-wide CRISPR screens were performed in the HLF cell line transduced with Cas9 expression vector lentiCas9-Blast (Addgene plasmid #52962) (*58*) and in the HLF clone HLF-LOH87 harboring chr8pLOH. Cells were cultured throughout the duration of the screen in 15-cm plates in DMEM supplemented with 10% FBS and 1% penicillin/streptomycin. To allow for a representation of 500 cells infected with a sgRNA, 2.46 × 10^6^ cells were transduced with the GeCKOv2 knockout library (*58*) at an aimed target multiplicity of infection lower than 0.25 as described above. Therefore, libraries A and B were pooled according to sgRNA representation. Cells were transferred to fresh plates 24 hours after addition of the virus and selected with puromycin (2 μg/ml) for additional 48 hours. On day 4 after infection, 61.5 × 10^6^ cells were collected by centrifugation representing a 500-fold coverage at time point 0. The same number of cells was passaged every 3 to 4 days for 14 more days and harvested at days 7 and 14. The screen was performed in three independent experimental replicates for each cell line. gDNA was extracted using the Zymo Quick-gDNA MidiPrep Kit (Zymo Research Europe, Freiburg, Germany). Library prep was performed as previously described (*74*) using the NEBNext Ultra II Q5 Polymerase (New England Biolabs, Frankfurt, Germany) and purified with AMPure Beads (Beckmann Coulter, Krefeld, Germany). Necessary adaptor sequences were attached by PCR reamplification, and amplicon purification was performed with NEB Monarch Polymerase (New England Biolabs, Frankfurt, Germany). The Illumina NextSeq 550 (Illumina, San Diego, CA, USA) was used for sequencing of pooled samples.

Reads from the CRISPR-Cas9 knockout screen were trimmed to the sgRNA sequence of amplicons using the tool cutadapt (*75*). The trimmed reads were mapped to the GeCKOv2 library using MAGeCK (*76*). To compensate for the batch effects, the raw counts were adjusted using Combat-Seq (*77*), which is part of the R package surrogate variable analysis (*78*). The batch-corrected reads were then analyzed using MAGeCK MLE in pairwise comparisons between the different conditions to determine gene essentialities (*79*). Genes with a waldFDR of <0.05 were considered to be significant. Normalized read counts and calculated gene essentiality scores (β scores calculated by MAGeCK) for all genes are provided in data S3. The raw data are deposited in the Sequence Read Archive (www.ncbi.nlm.nih.gov/sra/; project ID PRJNA906106; run IDs SRR22429880 to SRR22429895).

### In silico analysis of gene dependencies

For the in silico analysis of gene dependencies, the 20Q4 ACHILLES gene dependency and CCLE gene copy number datasets from DepMap database (https://depmap.org/portal/) were used (*24*). The datasets were broken down to all cell lines with both gene dependency data and copy number data available. For each cell line, a chromosome chr8pLOH copy number score was calculated as the mean copy number of all chr8p genes. Cell lines with a score of <0.7 were defined as chr8pLOH, a score of >0.825 and <1.175 was considered as chr8pWT. Limma analysis was performed comparing chr8pWT and chr8pLOH groups. For target identification, genes with *P* < 0.2 and more essential in the chr8pLOH group were overlapped between liver cancer analysis, pan-cancer analysis, and CRISPR screening data (fig. S5B).

### Lentiviral gene knockout

For validation of CRISPR screen hits, independent single guides were designed using chopchop.cbu.uib.no and cloned into the lentiGuide-Puro backbone (Addgene plasmid #52963) (*58*) using a one-step digestion-ligation reaction with the Esp 3I restriction enzyme. Lentiviral particles were generated, and cells were infected as described above. One day after infection, puromycin (1 μg/ml) was added for 2 to 3 days to select the cells. Knockout efficiency was validated by inference of CRISPR edits (ICE) analysis on the tide.nki.nl website or by Western blot. Two sgRNAs yielding the highest knockout efficiency were used for further validation experiments.

### Cell viability assay

For analysis of cell viability, 10,000–25,000 HLF, HLE, or HCC68 cells were seeded in 12-well plates in triplicates after respective lentiviral target gene knockout or siRNA-mediated knockdown. Then, 10% resazurin (R&D Systems, Minneapolis, MN, USA) diluted in growth medium was added to the cells and incubated for 1 hour at 37°C. Absorbance was measured at 544-nm excitation/590-nm emission using the Omega FLUOstar Microplate Reader (BMG LABTECH, Ortenberg, Germany) every 24 hours for 4 to 7 days.

### Colony formation assay

Gene dependency validation by colony formation was performed by seeding 1000 cells in six-well plates after lentiviral single and double target gene knockout or after siRNA-mediated knockdown, respectively. Cells were cultured for 14 days, washed with PBS, and stained with 0.5% crystal violet solution (Sigma-Aldrich, Taufkirchen, Germany) in 25% methanol (Honeywell, Charlotte, NC, USA) for 30 min at room temperature. Colony area was quantified using the Fiji software and normalized to the mean area of the controls.

### Immunofluorescence

For immunofluorescence, cells were seeded on glass cover slips and treated with H_2_O_2_ (10 μM) or left untreated for 1 hour. Cells were fixed in methanol/acetone for 10 min and permeabilized with 0.2% Triton X-100/PBS for 10 min. The primary antibody was incubated in a humid chamber at room temperature for 1 hour. Alexa Fluor 488 donkey anti-mouse secondary antibody (Jackson ImmunoResearch, Cambridgeshire, UK) was used at room temperature for 1 hour. DAPI Flouromount-G (Southern Biotech, Birmingham, USA) was used to mount cover slips. Images were taken using a Nikon AR1 confocal microscope and processed with Fiji software.

### Cell cycle analysis

For analysis of cell cycle distribution, a dual staining was performed combining the Click-iT EdU Alexa Fluor 488 Flow Cytometry Assay Kit (Thermo Fisher Scientific, Waltham, MA, USA) with FxCycle FarRed Stain (Thermo Fisher Scientific, Waltham, MA, USA). Thereby, cells were starved for 1 hour and treated with 5 μM H_2_O_2_ for an additional hour in FBS-free medium. EdU (10 μM) was added to the medium for DNA incorporation of proliferative cells. Cells were trypsinized, washed with PBS, and subsequently fixed and stained according to the manufacturer’s protocol. EdU incorporation and DNA amount were determined by flow cytometry using the Guava easyCite HT system (Merck Millipore, Darmstadt, Germany).

### β-Galactosidase staining

Senescent cells were visualized using staining by β-galactosidase. Cells were seeded on coverslips and washed with PBS and fixed with glutaraldehyde for 15 min after respective treatment. X-Gal staining solution (Roche, Mannheim, Germany) was added and incubated for 16 hours at 37°C. Coverslips were mounted on glass slides and imaged using an Olympus BX53 microscope with a 10× objective, and stained area was quantified in five to eight images for each experiment using the Fiji software.

### Plasmid cloning

#### pX458-tRNA–2 Mbp–tRNA–35 Mbp–dCas9-Puro

For transient sgRNA delivery, the pX458 vector (Addgene plasmid #48138) (*80*) was edited by first exchanging the U6 promoter with tRNA promoter, followed by introduction of a puromycin resistance instead of the *EGFP* gene and removal of the *Cas9* gene. The sgRNA sequences targeting chr8p regions were introduced via the Bbs I sites in independent vectors and then combined in the final vector.

#### pDEST26-gene-HA

*MSRA*, *NAT1*, *PPP2CB*, and *NUDT18* cDNA constructs were received from Stefan Pusch in the pDONR201 vector backbone. Gateway cloning in MACH1 *Escherichia coli* using the LR-Clonase was performed to transfer the genes into pDest-GW-HA destination vector.

#### lentiGuide-Puro-sgRNA

Annealing and phosphorylation of oligos for each sgRNA with respective overhangs were followed by a one-step digestion-ligation approach using the Esp 3I enzyme and the lentiGuide-Puro backbone (Addgene plasmid #52962) (*58*).

#### pTRIPZ-NUDT18-FLAG-C

A single FLAG tag was integrated into pTRIPZ Gateway vector (Thermo Fisher Scientific, Waltham, MA, USA) by annealing and phosphorylating FLAG oligonucleotides with Mlu I overhangs. A one-step digestion-ligation approach was used to add FLAG tag and destroy Mlu I sites. Gateway cloning of pTRIPZ-GW-FLAG-C and pENTR201-NUDT18 using the LR-Clonase was performed to change the ccdb cassette to the *NUDT18* cDNA. Antibiotic resistances were exchanged by restriction enzyme cloning with Kpn I and Not I restriction sites.

All plasmid sequences were verified by Sanger sequencing.

### Bioinformatic analysis

Bioinformatic analysis of TCGA, LICA-FR, and DepMap data was performed with the statistical computing environment R (version 4.0.4, www.R-project.org/). TCGA data were extracted using the RTCGAToolbox package, and LICA-FR data were obtained from the ICGC portal (*23*). A chr8pLOH score was calculated for each patient or cell line as the mean copy number of all chr8p genes. Patients were clustered into chr8pWT (copy number < 0.1 and >−0.1) and chr8pLOH (copy number < −0.33) groups. Mutation, copy number, and gene expression data were plotted using the ComplexHeatmap or ggplot2 packages. Statistical analysis between two groups in R was performed using the limma package.

### Statistical analyses

GraphPad Prism 9 (GraphPad Software, La Jolla, CA, USA) and the statistic software R were used for statistical analyses. Data are presented as means ± SD. Student’s *t* test was used for comparison of two groups assuming normal distribution. Analysis of variance (ANOVA) test was performed for multiple group comparisons using Sidak’s multiple comparison test. For Kaplan-Meier curves, log-rank test was performed. *P* values below 0.05 were considered statistically significant. Each figure legend denotes the statistical test used.
