# Supplementary material for: Chromosome 8p engineering reveals increased metastatic potential targetable by patient-specific synthetic lethality in liver cancer
Source: Sci Adv. 2023 Dec 22;9(51):eadh1442. doi: 10.1126/sciadv.adh1442 (PMC10745716; doi:10.1126/sciadv.adh1442)
Supplement: Supplementary file 1 — Figs. S1 to S8 Legends for data S1 to S5 [file sciadv.adh1442_sm.pdf]

Science Advances
AAAS

Supplementary Materials for

Chromosome 8p engineering reveals increased metastatic potential targetable by patient-specific synthetic lethality in liver cancer

Thorben Huth *et al.*

Corresponding author: Stephanie Roessler, stephanie.roessler@med.uni-heidelberg.de

Sci. Adv. **9**, eadh1442 (2023)
DOI: 10.1126/sciadv.adh1442

The PDF file includes:

Figs. S1 to S8
Legends for data S1 to S5

Other Supplementary Material for this manuscript includes the following:

Data S1 to S5

Fig. S1.

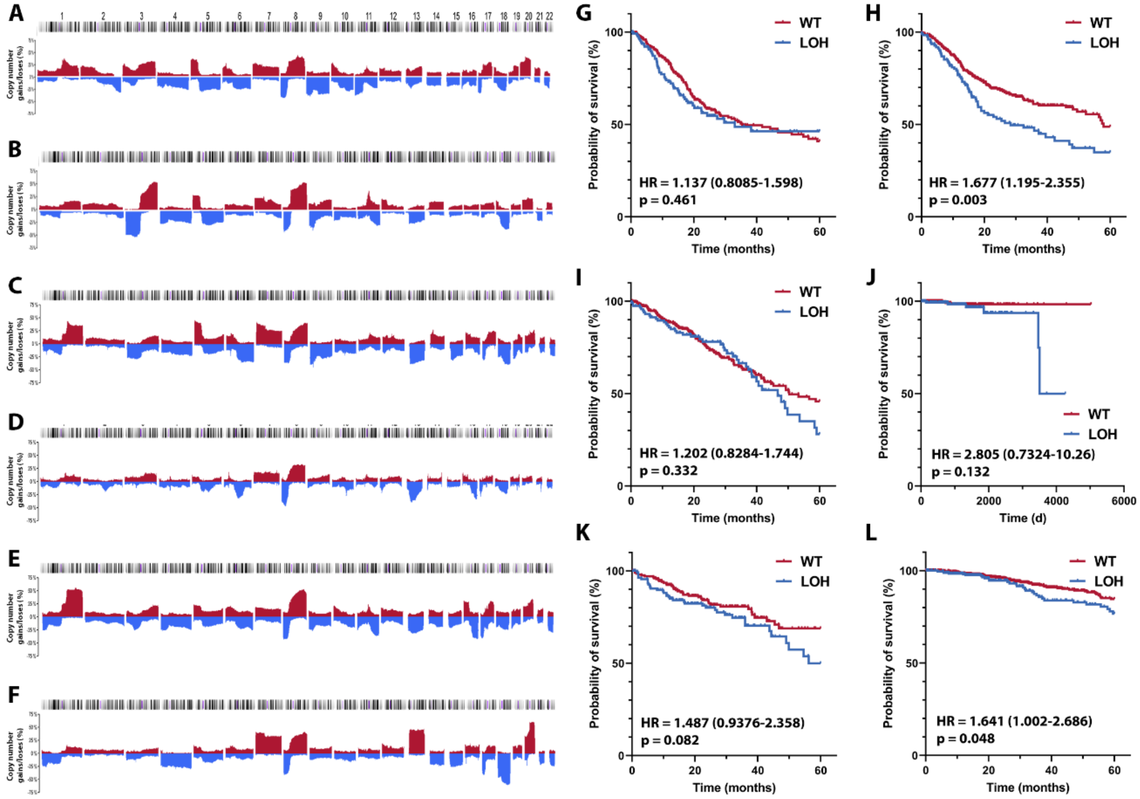

Figure S1: Chromosome 8p loss of heterozygosity (chr8pLOH) is observed in several cancer entities and associated with poor patient survival.

Copy number variation profiles in the TCGA cohort **(A)** BLCA, **(B)** HNSC, **(C)** LUAD, **(D)** PRAD, **(E)** BRCA and **(F)** COAD. Profile visualizations obtained from progenetix.org. Kaplan-Meier survival curves of the TCGA cohort **(G)** BLCA (WT: N=749, LOH: N=654), **(H)** HNSC (WT: N=305, LOH: N=124), **(I)** LUAD (WT: N=302, LOH: N=123), **(J)** PRAD (WT: N=306, LOH: N=167), **(K)** COAD (WT: N=220, LOH: N=142) and **(L)** BRCA (WT: N=599, LOH: N=282) clustered into chr8pWT (red) or chr8pLOH (blue) according to mean copy number. Hazard ration (HR) with 95% confidence interval and p-values were calculated by log-rank test.

Fig. S2.

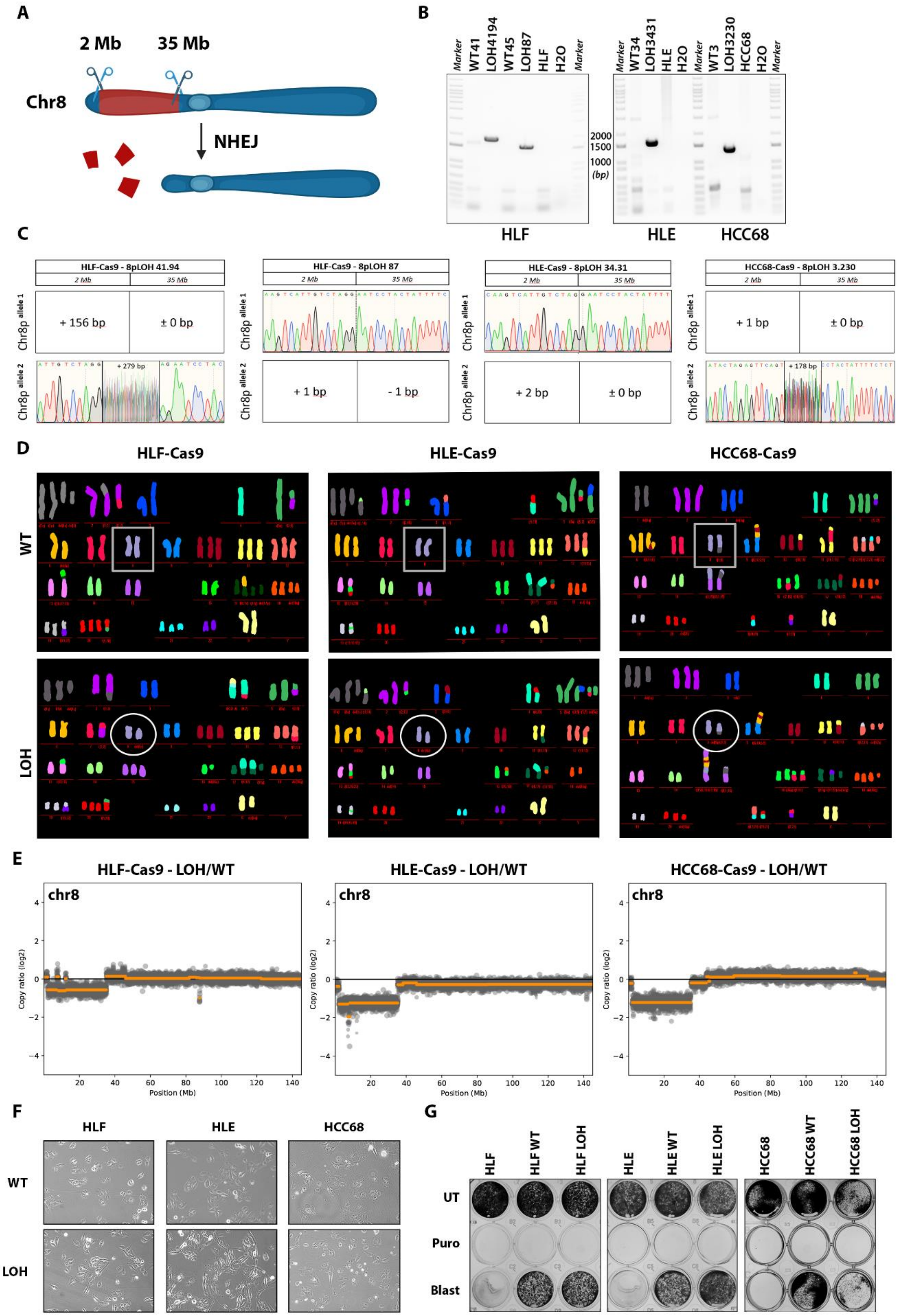

A
2 Mb
35 Mb
Chr8
NHEJ
B
Marker
WT41
LOH4194
WT45
LOH87
HLF
H2O
WT34
LOH3431
HLE
WT3
LOH3230
HCC68
2000
1500
1000
(bp)
HLF
HLE
HCC68
C
HLF-Cas9 - 8pLOH 41.94
HLF-Cas9 - 8pLOH 87
HLE-Cas9 - 8pLOH 34.31
HCC68-Cas9 - 8pLOH 3.230
Chr8p allele 1
Chr8p allele 2
+ 156 bp
± 0 bp
+ 279 bp
+ 1 bp
- 1 bp
+ 2 bp
+ 178 bp
D
HLF-Cas9
HLE-Cas9
HCC68-Cas9
WT
LOH
E
HLF-Cas9 - LOH/WT
HLE-Cas9 - LOH/WT
HCC68-Cas9 - LOH/WT
chr8
Copy ratio (log2)
Position (Mb)
F
HLF
HLE
HCC68
WT
LOH
G
HLF
HLF WT
HLF LOH
HLE
HLE WT
HLE LOH
HCC68
HCC68 WT
HCC68 LOH
UT
Puro
Blast

Figure S2: Screening and validation of chr8pLOH cell clones.

(A) Schematic illustration of dual-guided chromosome engineering using CRISPR/Cas9 technology. **(B)** Agarose gel images of the PCR products using primer pairs flanking the targeted region at 2 Mb and 35 Mb of chr8p. PCR product size is expected at 1500 bp or lower depending on the breakpoint repair. **(C)** Sanger sequencing results of PCR products using primer pairs flanking the targeted region of the deleted allele. Shown are the sequencing chromatograms. In addition, sequencing of the undeleted alleles by primers flanking each cut site (2 Mb and 35 Mb) revealed alterations, as indicated in the box, at each cut side individually. **(D)** Representative multiplex FISH of chr8pWT and chr8pLOH clones of HLF, HLE and HCC68 cells. Chromosome 8 is colored in light grey and highlighted with a white rectangle (WT) or circle (LOH). **(E)** WES-based paired copy number analysis of chr8 in HLF, HLE and HCC68 cells relative to their isogenic cell clone pair depicted as rainfall plots. **(F)** Brightfield microscopy images showing chr8pWT and chr8pLOH cell clone morphology in HLF, HLE and HCC68 cells. **(G)** Crystal violet stain of HLF, HLE and HCC68 cells before Cas9-Blast transduction and Cas9-expressing chr8pWT and chr8pLOH clones after selection with 2 µg/mL Puromycin (Puro) or 10 µg/mL Blasticidin (Blast).

Fig. S3.

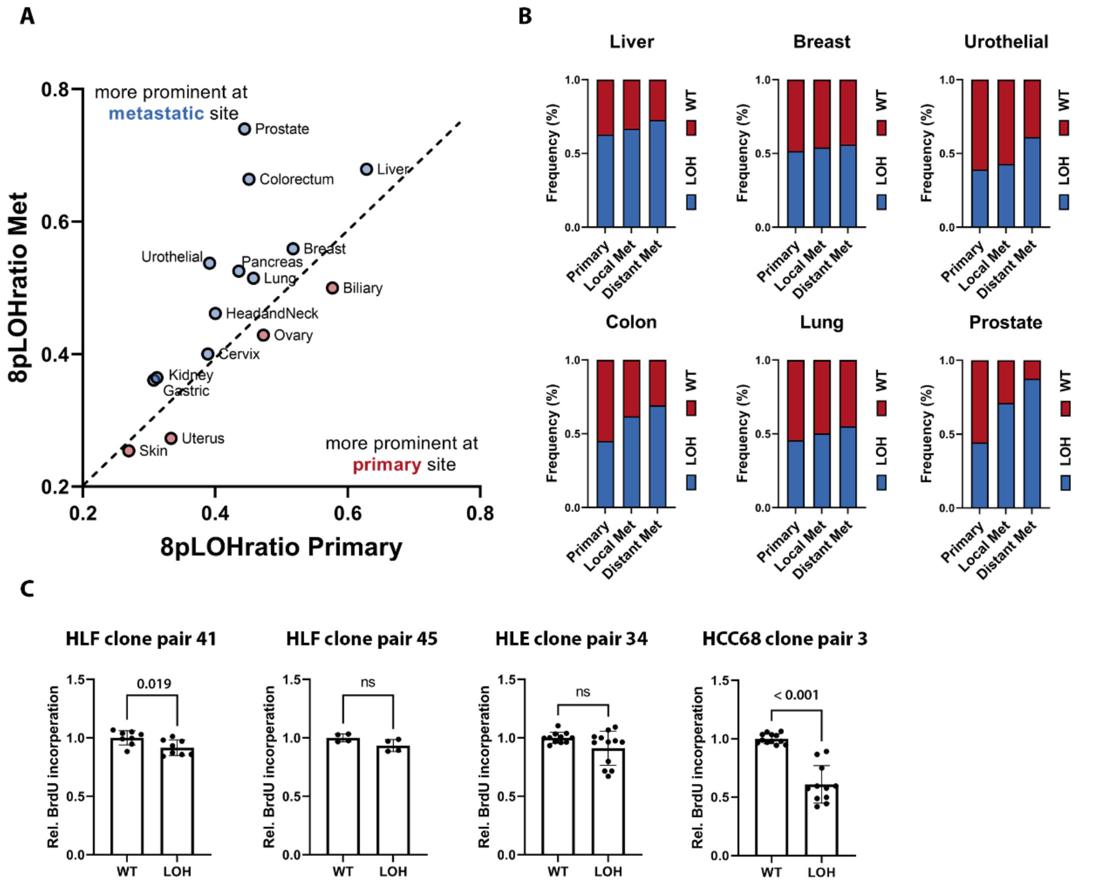

Figure S3: Chr8pLOH is associated with metastasis.

(A) Scatter plot showing ratios of patients with chr8p-deleted tumors at metastatic and primary sites. Data obtained from the Hartwig Medical Foundation dataset (*27*). **(B)** Frequency of patients with chr8pLOH or chr8pWT at primary site, local and distant metastases of selected cancer entities. **(C)** Proliferation of chr8pLOH cell clones determined by BrdU incorporation ELISA relative to respective chr8pWT clones. Data are represented as mean ± SD of two to three independent experiments. Single dots represent technical replicates. Student t-test was performed to determine p-values (p-value > 0.05, ns).

Fig. S4.

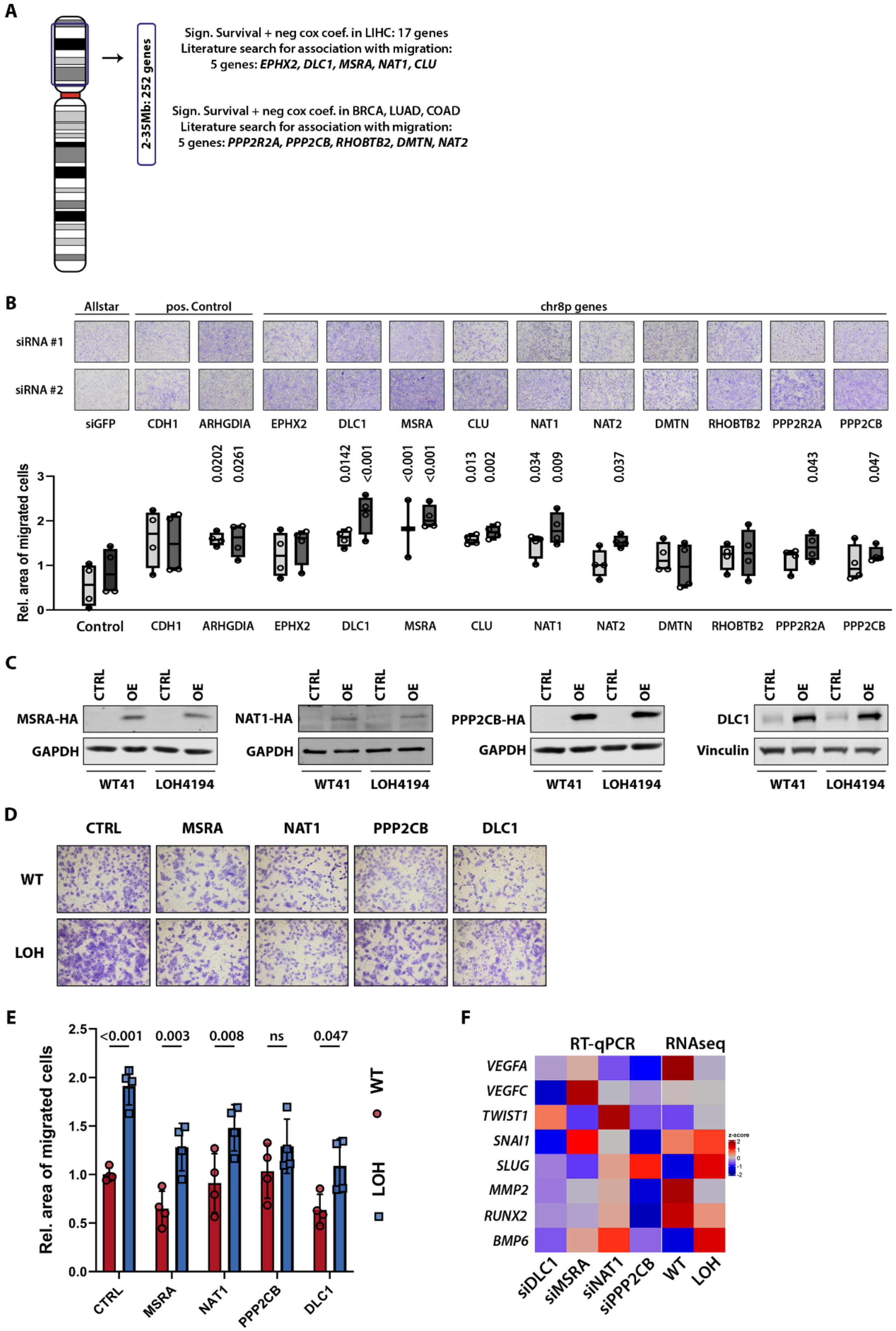
A
2-35Mb: 252 genes
Sign. Survival + neg cox coef. in LIHC: 17 genes
Literature search for association with migration:
5 genes: EPHX2, DLC1, MSRA, NAT1, CLU
Sign. Survival + neg cox coef. in BRCA, LUAD, COAD
Literature search for association with migration:
5 genes: PPP2R2A, PPP2CB, RHOBTB2, DMTN, NAT2
B
Allstar
pos. Control
chr8p genes
siRNA #1
siRNA #2
siGFP
CDH1
ARHGDIA
EPHX2
DLC1
MSRA
CLU
NAT1
NAT2
DMTN
RHOBTB2
PPP2R2A
PPP2CB
0.0202
0.0261
0.0142
<0.001
<0.001
<0.001
0.013
0.002
0.034
0.009
0.037
0.043
0.047
Rel. area of migrated cells
Control
C
CTRL
OE
MSRA-HA
GAPDH
NAT1-HA
PPP2CB-HA
DLC1
Vinculin
WT41
LOH4194
D
CTRL
MSRA
NAT1
PPP2CB
DLC1
WT
LOH
E
<0.001
0.003
0.008
ns
0.047
Rel. area of migrated cells
F
RT-qPCR
RNAseq
VEGFA
VEGFC
TWIST1
SNAI1
SLUG
MMP2
RUNX2
BMP6
z-score
siDLC1
siMSRA
siNAT1
siPPP2CB
WT
LOH

Figure S4: Scheme of metastasis suppressor screening approach and validation of metastasis-related genes on chr8p in HCC68 cells.

(A) Graphical scheme of identification of metastasis suppressor candidate genes on chr8p. **(B)** RNAi migration screen of chr8p candidate metastasis suppressors in HCC68 cells. Exemplary transwell migration images (top) are shown with respective quantification (bottom) of cell migration in four independent experiments. Knockdown was performed with two different siRNAs targeting each gene and quantified relative to Allstar and siGFP control (siRNA #1 – light grey, siRNA #2 – dark grey). Data are shown as floating bars with line indicating median and single dots representing each replicate of four independent experiments. **(C)** Western blot of HLF cell clones after transfection with empty vector (CTRL) or target gene overexpression (OE) and detection of HA tag and GAPDH or DLC1 and Vinculin. **(D)** Representative images of transwell migration assay in chr8pWT or chr8pLOH HCC68 cells after transfection with empty vector (CTRL) or target gene overexpression vectors (MSRA-HA, NAT1-HA, PPP2CB-HA, DLC1-V5). **(E)** Quantification of transwell migration in chr8pWT and chr8pLOH HCC68 cells after gene overexpression. Data are represented as mean ± SD of four independent experiments shown by single dots. **(F)** Heatmap of metastasis-associated gene expression after siRNA-mediated target gene knockdown in HCC68 cells compared to Allstar control (RT-qPCR data) and of chr8pWT and chr8pLOH HLF cells (RNAseq data). Z-scores are shown for gene expression relative to Allstar control (RT-qPCR) and relative to mean gene expression (RNAseq). Two-way ANOVA was performed for comparison of multiple groups. P-values are indicated above the graphs (p-value > 0.05, ns).

Fig. S5.

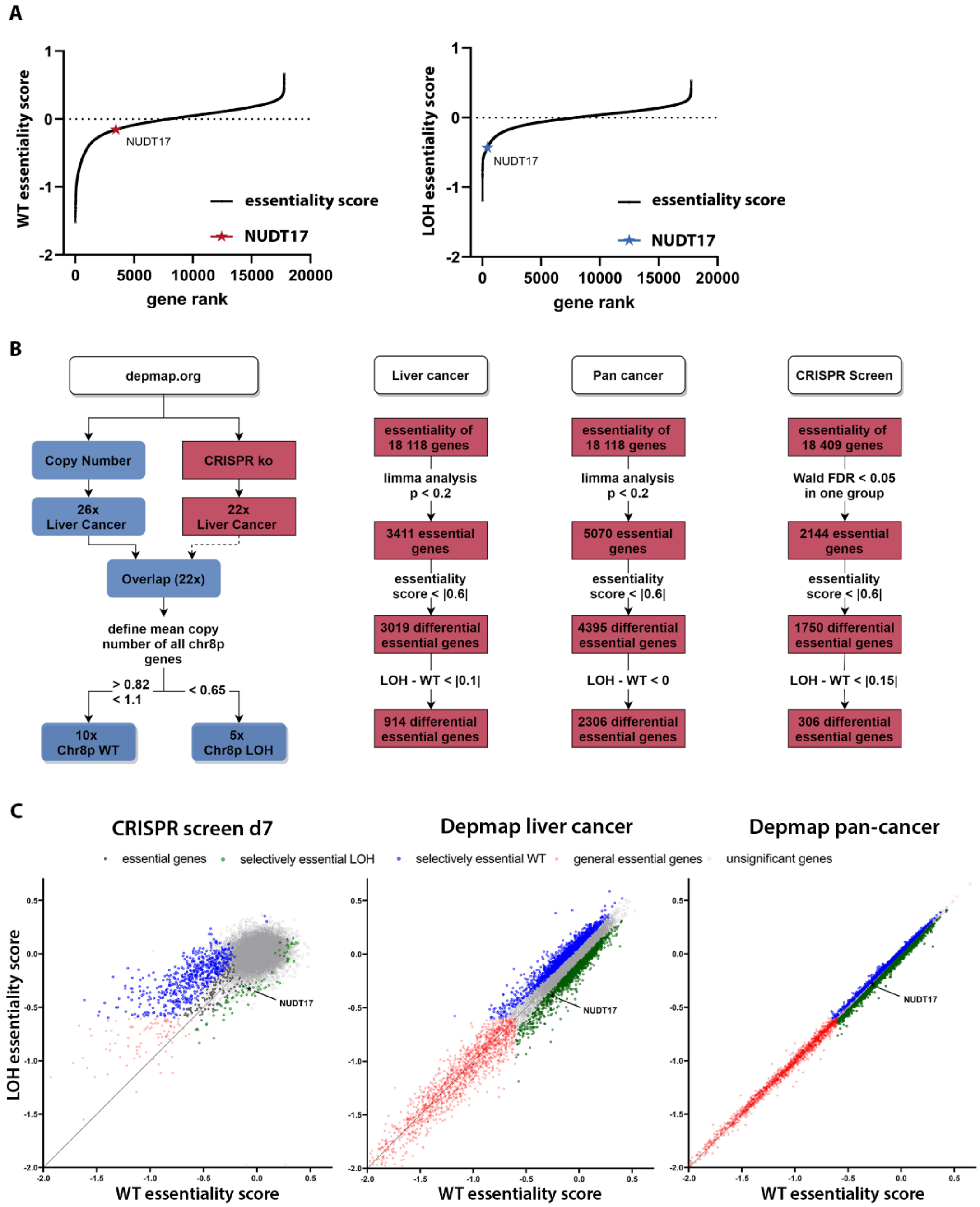

Figure S5: Extended data of the CRISPR/Cas9 knockout screen and the chr8pLOH DepMap analysis.

(A) Ranked gene essentiality scores of the CRISPR knockout screen in chr8pWT (left) and chr8pLOH (right) HLF cells. **(B)** Analysis flowcharts for the CRISPR knockout screen and DepMap analyses. **(C)** Scatter plot representation depicting gene essentiality scores in chr8pWT and chr8pLOH cells at day 7 (left) and mean gene essentiality scores of chr8pWT and chr8pLOH groups in the DepMap liver cancer (center) and pan-cancer (right) datasets.

Fig. S6.

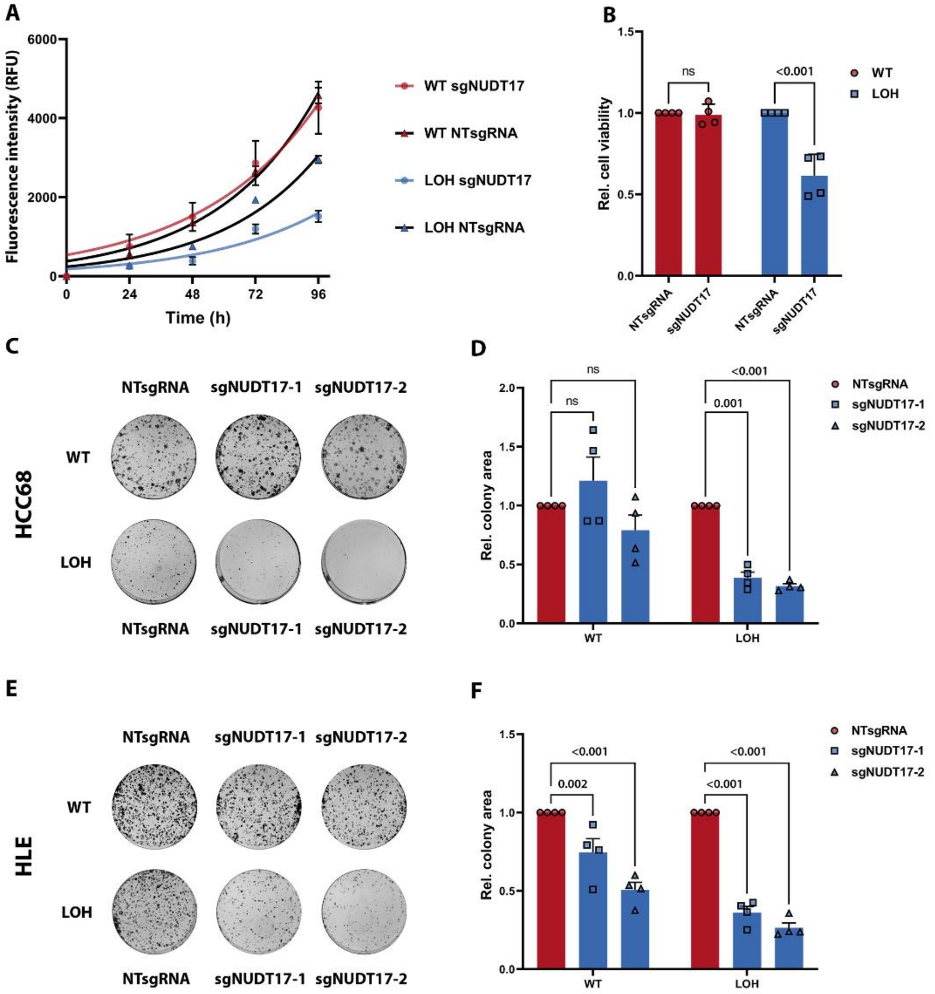

Figure S6: Validation of NUDT17 dependency in chr8pLOH cells.

(A) Growth curve for HCC68 chr8pWT and chr8pLOH cells after transduction with non-targeting sgRNA (NTsgRNA) or sgNUDT17 and cell viability measurement by resazurin assay for four consecutive days. Measurements of two independent sgRNAs for NUDT17 were combined. Out of four independent replicates, one representative growth curve is shown. Data are represented as mean ± SD of technical triplicates. **(B)** Relative cell viability of chr8pWT and chr8pLOH HCC68 cells following NUDT17 knockout 96 h post seeding. Data are represented as mean ± SD of four independent experiments with each dot representing the mean of one experiment. Colony formation of chr8pWT and chr8pLOH **(C-D)** HCC68 and **(E-F)** HLE cells. Cells were transduced with either NTsgRNA or two independent sgRNAs targeting NUDT17 and cultured for 14 days. Representative images of four replicates are shown. Quantification of colony area after NUDT17 knockout in chr8pWT and chr8pLOH **(D)** HCC68 and **(F)** HLE cells relative to NTsgRNA transduction. Data are represented as mean ± SD of four independent experiments with each dot representing the mean of one experiment. Two-way ANOVA was performed for comparison of multiple groups. P-values are indicated above the graphs (p-value > 0.05, ns).

Fig. S7.

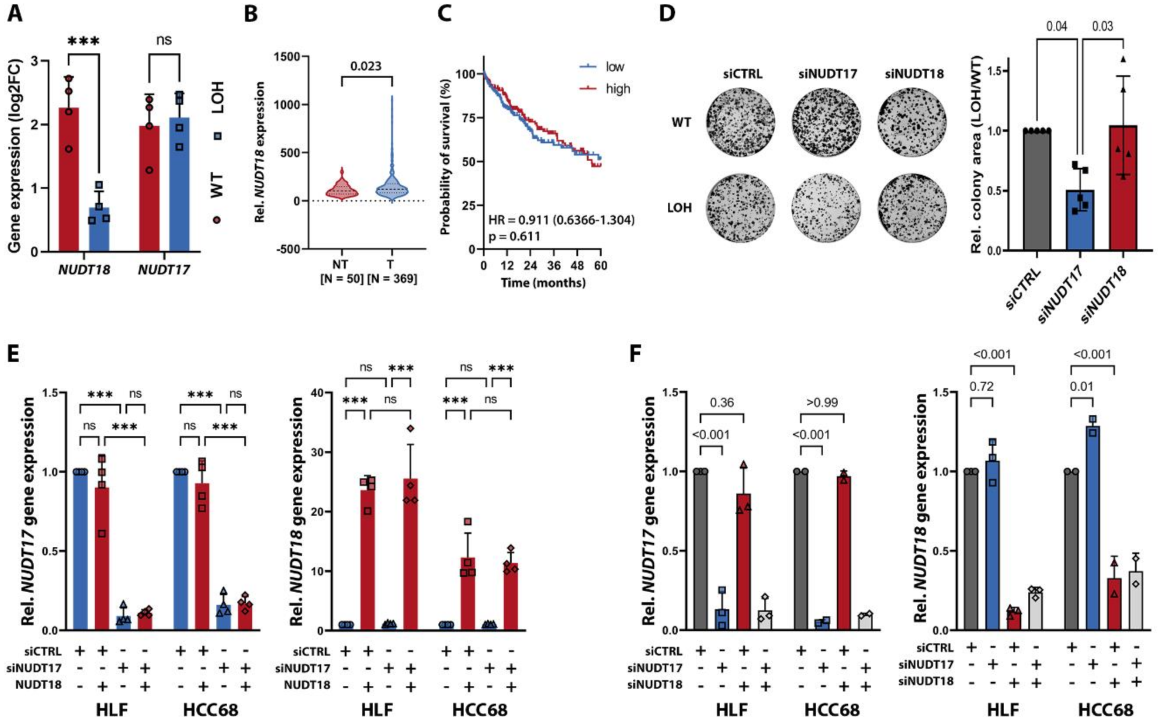

Figure S7: Analysis of NUDT17 and NUDT18 deregulation.

(A) NUDT17 and NUDT18 gene expression in four chr8pWT and four chr8pLOH cell clones as determined by RNAseq. **(B)** NUDT18 gene expression in TCGA-LIHC for normal liver tissue (NT) and HCC tumor (T) samples. **(C)** Kaplan-Meier survival curve of TCGA-LIHC patients with high (red, N = 185) or low (blue, N = 184) NUDT18 gene expression. Hazard ration (HR) with 95% confidence interval and p-values were calculated by log-rank test. **(D)** Colony formation of chr8pWT and chr8pLOH HLF cells. Cells were transfected with siPools targeting NUDT17, NUDT18 or negative control (siCTRL) and cultured for 14 days. Representative images of five replicates are shown. Quantification of colony area after NUDT17 and NUDT18 knockdown in chr8pWT and chr8pLOH cells relative to siCTRL. Data are represented as mean ± SD of five independent experiments with each dot representing the mean of one experiment. **(E)** NUDT17 and NUDT18 gene expression in pTRIPZ-NUDT18-infected chr8pLOH HLF and HCC68 cells after transfection with siNUDT17 or siCTRL and treatment with doxycycline (DOX). **(F)** NUDT17 and NUDT18 gene expression in chr8pWT HLF and HCC68 cells after siPool mediated knockdown of NUDT17 and NUDT18 alone or in combination. Gene expression was determined by quantitative RT-PCR and analyzed with the comparative Ct method. Two-way ANOVA was performed for comparison of multiple groups. P-values are indicated above the graphs (p-value > 0.05, ns; p < 0.001 ***).

Fig. S8.

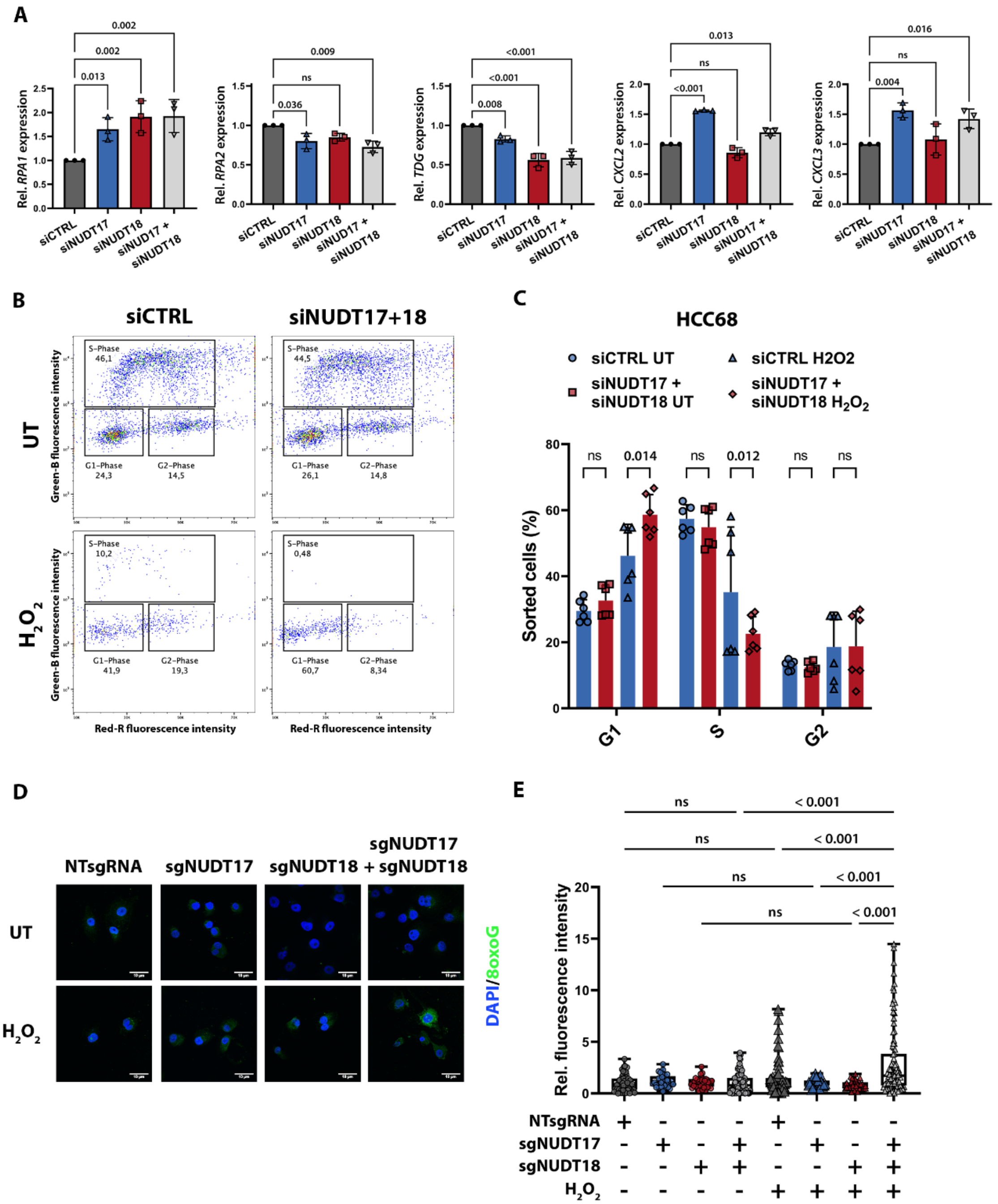

Figure S8: NUDT17 and NUDT18 loss affect cell cycle regulation, DNA damage response and senescence.

(A) Quantitative RT-PCR validation of senescence-, mismatch and base excision repair-associated gene expression (RPA1, RPA2, TDG, CXCL2, CXCL3) in HCC68 cells. **(B)** Scatter plot of flow cytometric analysis of HLF cells treated with H_2O_2 or left untreated (UT) and NUDT17/NUDT18 knockout after staining with EdU-Alexa488 (Green-B) and FxCycle FarRed (Red-R). Rectangular

gating and cell percentages for G1, S and G2 phases are shown. Images were generated using the FlowJo software. **(C)** HCC68 cell distribution in G1, S and G2 cell cycle phases after dual knockdown of NUDT17 and NUDT18 and treatment with 5 µM H_2O_2 or left untreated (UT). HCC68 cells were incubated with 10 µM EdU for 2 h to stain proliferative cells and DNA amount was visualized using FxCycle FarRed staining prior to flow cytometric measurement. **(D)** Immunofluorescence images of HCC68 cells after single and dual knockout of NUDT17 and NUDT18 and treatment with 10 µM H_2O_2. Nuclei were stained with DAPI in blue and cytosolic 8oxo-dGTP levels are shown in green. Knockout was performed with two independent sgRNAs for both genes. Exemplary images are shown for each condition. **(E)** Quantification of relative intensity of 8oxo-dGTP immunofluorescence. Data are represented as box-whisker plots with each dot representing one single cell out of 2-5 independent experiments. Image analysis was performed using Fiji software. Two-way ANOVA was performed for comparison of multiple groups. P-values are indicated above the graphs (p-value > 0.05, ns).

Other Supplementary Materials for this manuscript include the following:

Data S1. RNA sequencing data analyzed in this study

Data S2. Pathways analysis of differentially expressed genes

Data S3. Analysis results of the CRISPR-Cas9 screen and sgRNA distribution

Data S4: Oligonucleotide sequences and antibodies

Data S5: Mutations identified in the cell lines with or without chr8pLOH
